# Symptom-Region-Associated Neuromechanical Compensation During Running: Integrated 3D Kinematic, Kinetic and Electromyographic Profiling of Running-Related Injuries

**DOI:** 10.3390/jfmk11040378

**Published:** 2026-09-23

**Authors:** Ram Haddas, Brett P. Salazar, Ye Shu, Emily Hague, Jillian Santer, Edward M. Schwarz, Katherine H. Rizzone, Michael D. Maloney

**Affiliations:** Department of Orthopaedics, University of Rochester Medical Center, 601 Elmwood Ave, Box 665, Rochester, NY 14642, USA

**Keywords:** running-related injuries, running biomechanics, markerless motion capture, kinetic asymmetry, electromyography, neuromuscular control, rehabilitation

## Abstract

**Background and Objectives:** Running-related injuries (RRIs) are common musculoskeletal conditions associated with repetitive loading and heterogeneous biomechanical adaptations. This study evaluated the clinical utility of integrated three-dimensional (3D) biomechanical assessment for identifying interlimb asymmetries and symptom-region-specific compensatory strategies in runners with RRIs. **Methods:** One hundred twenty-two adolescent and adult runners referred from a Sports Medicine Physical Therapy clinic for a running-related musculoskeletal complaint were classified by primary symptomatic region (spine, n = 10; hip, n = 38; knee, n = 48; foot/ankle, n = 26); 27 asymptomatic runners served as controls. Participants completed standardized treadmill running while bilateral markerless 3D kinematics, instrumented treadmill kinetics, and surface electromyography (sEMG) were synchronously recorded. Log-transformed asymmetry indices and neuromechanical outcomes were evaluated using mixed-effects models with false discovery rate correction. **Results:** Distinct symptom-region-associated patterns of mechanical and neuromuscular compensation were identified. Hip-region RRIs demonstrated greater contralateral propulsive impulse (diff = 0.07; *p* = 0.029), whereas knee-region RRIs demonstrated greater contralateral hip extension moment (diff = 0.14; *p* = 0.013). Foot/ankle-region RRIs demonstrated greater contralateral knee extension moment (diff = 0.18; *p* = 0.017) and greater symptomatic-limb tibialis anterior activation (peak RMS: diff = 0.52; *p* = 0.032). The spine-region cohort demonstrated greater dominant-limb medial gastrocnemius activation (peak RMS: diff = 0.35; *p* = 0.037). **Conclusions:** Integrated 3D kinematic, kinetic, and neuromuscular profiling identified distinct regional patterns of load redistribution across the kinetic chain in runners with RRIs. This multimodal framework may complement clinical examination by identifying individualized biomechanical targets for rehabilitation and enabling longitudinal assessment of load-sharing recovery.

## 1. Introduction

Running-related injuries (RRIs) comprise load-related musculoskeletal conditions affecting bone, tendon, muscle, and joint structures, predominantly in the lower extremities [1,2]. They remain among the most common problems encountered in sports medicine and affect runners across age groups and levels of experience [3,4]. Reported incidence ranges from 15% to 63%, and many injuries disrupt training continuity, restrict participation, and produce persistent functional consequences [5,6]. Recurrence across running populations underscores an important clinical question: whether symptom resolution is accompanied by restoration of normal load sharing and movement strategy [7]. Although preventive exercise can reduce some running-related overuse injuries [8,9], current evidence does not support a single biomechanical risk profile or intervention that applies uniformly across runners [10,11,12]. This heterogeneity supports a shift from isolated impairment screening toward objective characterization of how an individual runner distributes mechanical and neuromuscular demand.

Running exposes the lower extremities to repeated mechanical loading, with vertical ground reaction forces commonly reaching approximately two to three times body weight [13]. The clinical consequence of this loading depends not only on force magnitude but also on how force is absorbed, transferred, and generated across the kinetic chain. Segmental alignment, joint motion, muscular capacity, neuromuscular control, and tissue load tolerance interact to determine how repetitive mechanical demand is distributed across the kinetic chain [14,15,16]. When load tolerance is impaired, runners may preserve task performance by altering motion at adjacent segments, shifting propulsion to the contralateral limb, or modifying muscle recruitment. Such compensation may be clinically important even when gross running form appears acceptable. Because running mechanics are modifiable through gait retraining [17,18], identifying the specific strategy used to maintain running may provide actionable targets for rehabilitation.

Three-dimensional (3D) motion capture, synchronized force measurement, and surface electromyography (sEMG) provide complementary information about this compensation: kinematics describe movement, kinetics quantify external and joint-level mechanical demand, and sEMG characterizes the neuromuscular strategy used to produce and control that movement [15]. Prior literature demonstrates substantial heterogeneity in biomechanical features associated with RRIs, suggesting that clinically meaningful dysfunction may be expressed as a distributed pattern rather than a single abnormal variable [10,11,19]. An integrated assessment may therefore be more informative than isolated kinematic measures, particularly when the symptomatic segment is unloaded through proximal, distal, or contralateral adaptations.

Two-dimensional (2D) video remains useful in clinical running assessment because it is accessible and time-efficient [20], but it cannot directly quantify multiplanar joint kinetics, ground reaction forces, mechanical work, or muscle activation. These limitations are relevant when the primary abnormality is redistribution of demand rather than an obvious movement deviation. Accordingly, the primary objective of this study was to determine whether a clinically implementable multimodal framework integrating markerless 3D kinematics, instrumented treadmill kinetics, and sEMG could identify interlimb mechanical and neuromuscular compensation in runners with RRIs. The secondary objective was to determine whether these compensation patterns differed according to the primary anatomical region of symptoms (spine, hip, knee, or foot/ankle). We hypothesized that injured runners would demonstrate symptom-region-associated asymmetry profiles spanning kinematic, kinetic, and neuromuscular domains rather than a uniform pattern of interlimb dysfunction.

## 2. Materials and Methods

### 2.1. Study Design

This study was reviewed by the University of Rochester Research Subjects Review Board and determined to meet federal and University criteria for exemption on 7 April 2025 (STUDY00010160; Exempt Category 4). The study is reported in accordance with the Strengthening the Reporting of Observational Studies in Epidemiology (STROBE) recommendations. The study was designed as an exploratory, hypothesis-generating evaluation of the clinical utility of integrated biomechanical profiling. Because the analysis used an available clinical cohort and was exploratory in nature, no a priori power analysis was performed.

### 2.2. Participants

A total of 122 runners with clinically evaluated RRIs (spine, n = 10; hip, n = 38; knee, n = 48; foot/ankle, n = 26) and 27 asymptomatic controls were included (Table 1). Demographic and running characteristics were recorded to contextualize biomechanical findings and inform covariate modeling. No additional participants were excluded from the analyzed cohort because of incomplete or technically unusable data.

### 2.3. Eligibility Criteria and Clinical Classification

*Clinical RRI cohort:* Participants were eligible for the clinical cohort if they were runners referred from a Sports Medicine Physical Therapy clinic for biomechanical evaluation of an active musculoskeletal complaint associated with running. No uniform minimum symptom duration, pain-severity threshold, imaging requirement, or diagnosis-specific criterion was imposed because eligibility reflected referral for clinically indicated running assessment. The complaint was required to involve the spine or lower extremity and to be clinically relevant to running participation, tolerance, or function. Participants were classified according to the primary anatomical region identified in the referral and clinical record as the principal site of the running-related complaint.

*Clinical classification:* Participants represented a real-world sports-medicine referral population with a heterogeneous spectrum of common running-related musculoskeletal conditions. Cohort assignment was based on the primary symptomatic anatomical region documented in the referral and available clinical record rather than a specific pathoanatomic diagnosis. Imaging and prior treatment were considered components of routine clinical care when available but were not required for cohort assignment.

*Control cohort:* Asymptomatic controls were runners without a current musculoskeletal complaint affecting running at the time of biomechanical testing. Controls underwent the same standardized running-assessment protocol as the clinical cohorts.

### 2.4. Instrumentation

Kinematic data were captured during running using an eight-camera markerless motion-analysis system (FLIR; Vicon Motion Systems Ltd., Oxford, UK) at 100 Hz. Ground reaction forces were recorded at 2000 Hz using a fully instrumented dual-belt treadmill (ITC-21-20 Standard Treadmill, Bertec Corporation, Columbus, OH, USA; Figure 1). Force sensors were zeroed before each session. Muscle activation was recorded using a wireless surface electromyography system (Trigno, Delsys Inc., Boston, MA, USA). Markerless motion-capture data were processed in Theia3D (version 2024.2; Theia Markerless Inc., Kingston, ON, Canada), a deep-learning-based system that has demonstrated generally good agreement and repeatability relative to marker-based approaches for gait and running applications [21,22,23,24].

### 2.5. Preparation and Testing Procedures

Before testing, motion-capture and force systems were calibrated according to manufacturer procedures, with a custom L-frame defining the laboratory coordinate system. Age, sex, body mass index, foot dominance, and running experience were recorded (Table 1). Participants wore habitual running shoes and orthoses when routinely used. Wireless sEMG electrodes were placed bilaterally over the tibialis anterior, medial gastrocnemius, rectus femoris, and semitendinosus according to standardized SENIAM-based anatomical placement procedures [19]. Maximum voluntary isometric contractions (MVCs) were obtained in triplicate for normalization, with 5 s contractions separated by 3 s rest periods. Muscle-specific standardized positions were used for dorsiflexion, plantarflexion, knee flexion, and quadriceps activation, and electrodes were secured to minimize movement artifact. Participants then completed a 10 min treadmill protocol at 0% incline. After a 3 min accommodation period at a self-selected running speed, a continuous 1 min steady-state window was recorded for analysis (Figure 1).

### 2.6. Data Acquisition and Processing

Gait events were identified using synchronized positional and force data. The force plate data were resampled to 100 Hz. Heel strike was defined after the foot reached its maximum anterior position and vertical force exceeded 150 N; toe-off occurred when force fell below 100 N. Midstance was defined by the anterior–posterior ground reaction force transition from braking to propulsion. A gait cycle was defined from heel strike to subsequent ipsilateral heel strike. Markerless kinematic data were processed in Theia3D, and filtered at 6 Hz using a generalized cross-validation spline (GCVSPL) filter. The processed data were exported in C3D format for synchronization with force plate and sEMG signals in Vicon Nexus 2.20.1. Segment transformations were analyzed using a biomechanical model in Vicon ProCalc 1.6.1. Trunk, pelvis, hip, knee, ankle, shank-to-vertical angle, and foot kinematics were extracted at heel strike, midstance, and toe-off, with range of motion calculated across each stride. Center-of-mass excursion and heel-to-center-of-mass projection measures were derived from segment-based anthropometric estimates. Joint kinetics were calculated through inverse dynamics by combining Theia kinematics with synchronized ground reaction force data in Vicon ProCalc.

Kinetic and sEMG signals were sampled at 2000 Hz and synchronized with kinematics. EMG signals were band-pass filtered at 20–450 Hz, converted to root mean square (RMS) amplitude using a 100-sample sliding window, and normalized to participant-specific MVC values. Activation was defined when normalized RMS exceeded 10% of the smoothed maximum. Outcomes included peak RMS magnitude and timing, onset timing, percentage of the gait cycle active, and integrated RMS. Triplicate MVC trials were averaged for normalization. Ground reaction forces were normalized to body weight. Joint moments were calculated in ProCalc, angular velocity was obtained by differentiating joint angles, and joint power was calculated as normalized moment multiplied by angular velocity. Work was obtained by integrating power over loading (heel strike to midstance) and propulsion (midstance to toe-off). Vertical-force outcomes included stance time, vertical impulse, impact peak, loading rate, and peak stance force. Anterior–posterior force was smoothed with a 100-point moving-average filter; braking and propulsive phases were defined from zero crossings, and braking, propulsive, and net impulses were calculated by numerical integration (Supplementary Table S2).

### 2.7. Outcome Measures

Three-dimensional kinematics, kinetics, and sEMG were collected synchronously using standardized laboratory procedures. Outcomes included joint and segment angles, joint moments and powers, ground reaction force variables, spatiotemporal measures, and muscle-activation characteristics. Variables were normalized where appropriate to body mass or MVC. Interlimb asymmetry was quantified using a log-transformed asymmetry index, AI = log(symptomatic/asymptomatic). For the spine and asymptomatic control cohort, the asymmetry index was calculated as AI = log(dominant/nondominant); therefore, positive values indicate greater magnitude on the dominant limb, and negative values indicate greater magnitude on the nondominant limb. Because the spine cohort was classified by axial symptoms, these values should not be interpreted as symptomatic versus asymptomatic limb differences. For the hip, knee, and foot/ankle cohorts, the symptomatic limb corresponded to the side of the primary clinically identified running-related complaint; the opposite extremity was designated the contralateral limb. Positive values indicate a greater magnitude on the symptomatic limb, whereas negative values indicate a greater magnitude on the contralateral limb. AI was calculated at the individual gait cycle.

### 2.8. Statistical Analysis

Comparisons between each RRI subgroup (spine, hip, knee, and foot/ankle) and asymptomatic controls were performed using mixed-effects linear regression models in R (v2025.5.1.513). Age and running level were included as fixed effects. Gait cycles were analyzed at the trial level, with random intercepts for participants and trials nested within participants to account for repeated gait cycles and within-participant correlation. On average 161 gait cycles were included per participant. Model fit was evaluated using Akaike and Bayesian information criteria. Because a large number of biomechanical outcomes were examined, variables were prespecified into biomechanically coherent families (spatiotemporal, kinematic, kinetic, and neuromuscular domains), and the Benjamini–Hochberg procedure was applied within each family to control the false discovery rate. All reported *p*-values are FDR-adjusted; a two-sided adjusted *p* < 0.05 was considered statistically significant. Given the exploratory design and unequal subgroup sizes, findings were interpreted as hypothesis-generating rather than confirmatory.

## 3. Results

### 3.1. Participant Characteristics

The analysis included 122 clinically referred runners with RRIs classified according to the primary symptomatic anatomical region: spine (n = 10), hip (n = 38), knee (n = 48), and foot/ankle (n = 26), and 27 asymptomatic controls. Each RRI cohort represented a heterogeneous sports-medicine population encompassing common musculoskeletal diagnoses within the respective anatomical region rather than a single diagnosis-specific condition. Participant characteristics are summarized in Table 1. No statistically significant between-group differences were identified for age, body mass index, height, body mass, cadence, stride length, or self-selected running speed (all *p* > 0.05). Principal symptom-region-associated neuromechanical findings are summarized in Table 2. Complete neuromuscular, kinetic, and kinematic results are provided in Supplementary Tables S1–S3, respectively. Sagittal-plane lower-extremity joint moment profiles and representative neuromuscular activation timing patterns across RRI cohorts are presented in Figure 2 and Figure 3, respectively.

### 3.2. Spine-Region RRI Cohort

Runners with a primary spine-region RRI demonstrated dominant-to-nondominant limb differences spanning kinematic, kinetic, and neuromuscular domains (Supplementary Tables S1–S3). Kinematically, the spine-region cohort demonstrated significant distal interlimb differences involving sagittal ankle motion, tibial inclination, and foot motion (Supplementary Table S3). Kinetically, stance duration was greater on the dominant limb across all gait phases (diff = 0.02, 95% CI 0.01 to 0.04; *p* = 0.010). No significant differences were identified in loading response, midstance moments, propulsive work, or vertical and forward force plate variables. Neuromuscularly, medial gastrocnemius activity was elevated on the dominant limb, as reflected by a higher peak RMS (diff = 0.35, 95% CI 0.02 to 0.67; *p* = 0.037) and greater integrated RMS (diff = 0.32, 95% CI 0.02 to 0.62; *p* = 0.037). No significant asymmetries were identified in tibialis anterior, rectus femoris, or semitendinosus activation across MVC voltage, onset timing, peak RMS timing, or usage.

### 3.3. Hip-Region RRI Cohort

Runners with a primary hip-region RRI demonstrated symptom-region-associated interlimb differences spanning kinematic, kinetic, and neuromuscular domains (Table 2; Supplementary Tables S1–S3). Kinematically, the hip-region cohort demonstrated significant interlimb differences in pelvic and hip motion, including sagittal pelvic and hip measures at midstance and coronal/transverse hip measures (Supplementary Table S3). Kinetically, total stance loading was increased (diff = 0.02, 95% CI 0.00 to 0.03; *p* = 0.050). During loading response, peak hip extension moment was not significantly different between limbs. At midstance, knee extension moment was greater on the contralateral limb (diff = 0.14, 95% CI 0.01 to 0.28; *p* = 0.035). During the propulsive phase, positive hip work (diff = 0.27, 95% CI 0.01 to 0.53; *p* = 0.040) and positive ankle work (diff = 0.08, 95% CI 0.01 to 0.15; *p* = 0.019) were increased on the contralateral limb. Propulsive impulse was likewise greater on the contralateral limb (diff = 0.07, 95% CI 0.01 to 0.13; *p* = 0.029). Neuromuscularly, tibialis anterior peak RMS was increased on the symptomatic limb (diff = 0.47, 95% CI 0.01 to 0.94; *p* = 0.046), and integrated RMS was greater (diff = 0.38, 95% CI 0.07 to 0.70; *p* = 0.018). No significant differences were identified in onset timing, peak RMS timing, or usage for any of the four muscles.

### 3.4. Knee-Region RRI Cohort

Runners with a primary knee-region RRI demonstrated distributed asymmetry across gait phases, involving proximal and distal kinematics, propulsion, and neuromuscular timing. (Supplementary Tables S1–S3). Kinematically, at initial contact, sagittal pelvic motion was reduced on the symptomatic side (diff = −1.16, 95% CI −1.88 to −0.44; *p* = 0.002), while coronal hip motion was increased (diff = 0.84, 95% CI 0.12 to 1.57; *p* = 0.023). During midstance, sagittal ankle motion (diff = 0.14, 95% CI 0.06 to 0.21; *p* = 0.001), sagittal foot motion (diff = −0.39, 95% CI −0.63 to −0.15; *p* = 0.002), and coronal hip motion (diff = 0.83, 95% CI 0.29 to 1.37; *p* = 0.003) were significantly asymmetric. At toe-off, sagittal pelvic motion was reduced (diff = −1.00, 95% CI −1.68 to −0.32; *p* = 0.005), while sagittal knee motion (diff = 0.15, 95% CI 0.01 to 0.28; *p* = 0.031) and coronal pelvic motion (diff = 0.24, 95% CI 0.06 to 0.42; *p* = 0.008) were increased. Range-of-motion asymmetries were present in sagittal tibial inclination (diff = 0.04, 95% CI 0.01 to 0.08; *p* = 0.014), coronal knee motion (diff = 0.20, 95% CI 0.05 to 0.36; *p* = 0.012), and coronal ankle motion (diff = 0.26, 95% CI 0.10 to 0.41; *p* = 0.001).

Kinetically, total stance loading was increased on the symptomatic limb (diff = 0.02, 95% CI 0.00 to 0.03; *p* = 0.050). During loading response, peak hip extension moment was greater on the contralateral limb (diff = 0.14, 95% CI 0.03 to 0.26; *p* = 0.013). Propulsive impulse was also greater on the contralateral limb (diff = 0.07, 95% CI 0.01 to 0.13; *p* = 0.031). No significant between-limb differences were identified in midstance ankle plantarflexion moment, midstance knee extension moment, propulsive hip or ankle work, or vertical and braking force plate metrics. Neuromuscularly, tibialis anterior MVC voltage was greater on the symptomatic limb (diff = 0.53, 95% CI 0.24 to 0.83; *p* = 0.001), and peak RMS was greater on the contralateral limb (diff = −0.49, 95% CI −0.91 to −0.07; *p* = 0.023). Peak RMS timing of the rectus femoris was earlier on the symptomatic limb (diff = −0.21, 95% CI −0.43 to 0.00; *p* = 0.048). Semitendinosus onset timing was delayed on the symptomatic side (diff = 0.77, 95% CI 0.06 to 1.47; *p* = 0.035), and peak RMS timing was earlier (diff = −0.15, 95% CI −0.30 to 0.00; *p* = 0.046).

### 3.5. Foot and Ankle-Region RRI Cohort

Runners with a primary foot/ankle-region RRI demonstrated a combined proximal–distal pattern consistent with compensation involving pelvic and hip kinematics, contralateral kinetic loading, and symptomatic-limb tibialis anterior activation (Supplementary Tables S1–S3). Kinematically, at initial contact, sagittal pelvic motion was reduced on the symptomatic side (diff = −1.21, 95% CI −2.11 to −0.31; *p* = 0.010), coronal hip motion was reduced (diff = −1.12, 95% CI −2.04 to −0.19; *p* = 0.019), and transverse foot progression was asymmetric (diff = −0.83, 95% CI −1.57 to −0.10; *p* = 0.027). During midstance, sagittal pelvic motion remained reduced (diff = −1.29, 95% CI −2.15 to −0.43; *p* = 0.004), while sagittal hip motion was increased on the symptomatic side (diff = 0.11, 95% CI 0.01 to 0.21; *p* = 0.035), with this asymmetry persisting at toe-off (diff = 0.11, 95% CI 0.00 to 0.22; *p* = 0.049). Trunk coronal range of motion was reduced on the symptomatic side (diff = −0.03, 95% CI −0.06 to −0.01; *p* = 0.020), and trunk transverse range of motion was asymmetric (diff = 0.00, 95% CI 0.00 to 0.01; *p* = 0.019).

Kinetically, total stance loading was increased on the contralateral limb (diff = 0.02, 95% CI 0.00 to 0.03; *p* = 0.028). Midstance knee extension moment was greater on the contralateral limb (diff = 0.18, 95% CI 0.03 to 0.33; *p* = 0.017). No significant between-limb differences were identified in loading response variables, propulsive work, or vertical and forward force plate metrics. Neuromuscularly, tibialis anterior peak RMS was increased on the symptomatic limb (diff = 0.52, 95% CI 0.05 to 1.00; *p* = 0.032), integrated RMS was greater (diff = 0.44, 95% CI 0.10 to 0.77; *p* = 0.013), and peak RMS timing was delayed (diff = 0.34, 95% CI 0.01 to 0.67; *p* = 0.041). No significant asymmetries were identified in medial gastrocnemius, rectus femoris, or semitendinosus activation.

## 4. Discussion

The principal finding of this study is that RRIs were not characterized by a single, uniform biomechanical asymmetry. Instead, the integrated assessment identified symptom-region-specific neuromechanical strategies that redistributed demand across limbs and segments while runners maintained the same functional task. Runners with a primary hip-region RRI shifted knee extension demand, positive hip and ankle work, and propulsive impulse toward the contralateral limb. Knee-related injuries similarly demonstrated greater contralateral hip extension moment and propulsive impulse, but this mechanical redistribution was accompanied by altered rectus femoris and semitendinosus timing. Runners with a primary foot/ankle-region RRI combined contralateral stance loading and knee extension demand with increased and delayed tibialis anterior activation on the symptomatic side. The spine-region cohort demonstrated a distinct distal kinematic and gastrocnemius pattern; however, this finding should be interpreted cautiously given the small subgroup size (n = 10) and corresponding reduced precision of the estimates. The spine-region RRI cohort demonstrated a different phenotype dominated by distal kinematic asymmetry and increased medial gastrocnemius activity. Importantly, interlimb asymmetry is also present in uninjured runners and therefore should not be interpreted as pathological in isolation [25]. Accordingly, the clinically relevant phenotype was not simply the presence of asymmetry, but the regional pattern, direction, and neuromuscular context of load redistribution associated with the runner’s primary clinical presentation.

This distinction is clinically important because symptom location does not necessarily identify where mechanical demand is being managed during running. In the hip and knee cohorts, greater contralateral propulsive demand was consistent with redistribution of selected functions away from the symptomatic limb. In the foot/ankle cohort, the combination of proximal kinematic adaptation, contralateral kinetic loading, and increased symptomatic-limb tibialis anterior activity illustrates a different strategy: local neuromuscular demand may increase even while selected mechanical demands are transferred elsewhere. These findings argue against interpreting an apparently normal or successful running trial as evidence of restored biomechanics. For sports-medicine clinicians, objective gait and loading data may complement examination and imaging by showing how the patient accomplishes the task, not simply whether the task can be completed [18,20]. The regional cohorts intentionally reflect the heterogeneity encountered in sports-medicine practice, consistent with evidence demonstrating substantial variability in biomechanical and clinical factors associated with RRIs [10,11,12,26]. Accordingly, the observed profiles should be interpreted as regional clinical phenotypes rather than diagnostic signatures of individual conditions.

The findings also refine how biomechanical data may be translated into rehabilitation. A runner with reduced symptomatic-limb propulsion may require a different intervention from a runner who preserves propulsion but demonstrates excessive distal muscle demand or proximal patterns consistent with compensation. Accordingly, treatment targets may be better selected from the individual neuromechanical phenotype than from injury location alone. Rehabilitation can then be directed toward the specific deficit identified, for example, restoring propulsive contribution, improving joint-specific load acceptance, addressing proximal control, or reducing excessive compensatory muscle demand, while continuing to evaluate the contralateral limb [17,18]. Serial testing may be particularly valuable when symptoms improve before load-sharing strategies normalize, providing an objective functional complement to pain scores and patient-reported outcomes [27]. Prospective studies are needed to establish responsiveness and clinically meaningful thresholds.

From a clinical implementation perspective, the value of this multimodal framework lies in integrating complementary measures rather than relying on any single biomechanical domain. Markerless motion capture offers a clinically practical alternative to conventional marker-based approaches [21,22,23,24], while synchronized force measurement and sEMG provide information that kinematics alone cannot capture. The resulting profile links what the runner does (kinematics), where mechanical demand is generated or absorbed (kinetics and work), and how the neuromuscular system supports that strategy (sEMG). This structure is well suited to clinical phenotyping because it converts a large biomechanical dataset into a mechanistic question: where is demand being redistributed, and what compensatory strategy permits continued running? Such information may ultimately support rehabilitation progression and return-to-running decisions [28]. However, the present cross-sectional study does not establish prospective injury risk, treatment responsiveness, or clearance thresholds. Interventions that modify movement strategy may also influence interlimb load distribution; however, symmetry should be interpreted alongside absolute joint loading, symptoms, and functional capacity rather than treated as an isolated rehabilitation endpoint.

Several limitations should be considered when interpreting these findings. First, the retrospective, cross-sectional design cannot determine whether the observed asymmetries preceded injury, emerged in response to symptoms, or represent adaptive strategies to altered load tolerance. The findings should therefore not be interpreted as causal risk factors. Second, subgroup sizes were unequal, particularly for the spine-region RRI cohort (n = 10), reducing precision for region-specific estimates. Third, participants were classified by primary symptomatic anatomical region rather than a specific pathoanatomic diagnosis, resulting in clinically heterogeneous cohorts representative of sports-medicine practice. Although this regional classification enhances clinical relevance, it limits diagnosis-specific interpretation of the observed biomechanical patterns. Symptom duration, pain severity, symptom laterality, and pain experienced during the recorded running trial were not uniformly captured across this retrospective clinical cohort. Accordingly, the present analysis cannot distinguish acute pain-related movement adaptations from more persistent neuromechanical characteristics. Fourth, Participants ran at self-selected speeds, which increased ecological validity but introduced potential speed-related biomechanical variability. Although the differences between group speeds were not statistically significant, the control group ran numerically faster than several RRI cohorts; therefore, residual speed-related effects cannot be excluded. Future studies should stratify participants by diagnosis, symptom severity and chronicity, and treatment status to identify more specific neuromechanical phenotypes. Fourth, self-selected treadmill speed and inclusion of both adolescent and adult runners increase ecological relevance but also introduce biomechanical variability; relevant covariates were incorporated into the models. Fifth, markerless motion capture and associated biomechanical modeling introduce measurement uncertainty relative to conventional marker-based approaches [21,22,23,24]. sEMG outcomes may likewise be influenced by electrode placement, normalization, and signal-processing procedures [19,29]. Finally, the instrumented treadmill and sEMG requirements may limit immediate adoption outside specialized motion-analysis settings. Prospective longitudinal studies should test whether the identified profiles change with symptom resolution, respond to targeted rehabilitation, predict recurrence, and define thresholds that are meaningful for clinical progression.

### Clinical Case Illustration

The following case illustrates the clinical application of multimodal biomechanical profiling in an individual runner and is presented as a translational example rather than as evidence of diagnostic or prognostic validity. A 17-year-old male varsity track and cross-country distance runner presented with several months of atraumatic right foot pain, localized to the metatarsals and occurring exclusively during running. He reported training predominantly on asphalt and road surfaces, averaging 40–50 miles per week, with no use of orthotics. His medical history was notable only for a remote right ankle sprain, and he had no prior surgical history. On examination, he demonstrated focal tenderness over the midshaft of the right metatarsals. MRI confirmed a midshaft second metatarsal stress reaction (Figure 4). He was withheld from running, permitted to continue cycling and swimming, and referred to physical therapy. After four months, he achieved clinical and radiographic resolution and successfully returned to sport following a structured return-to-running program. Two years later, during his collegiate career, he presented with new-onset left foot pain after completing a track season. Symptoms were similar in character and timing to his prior injury, but MRI findings were unremarkable. He returned to physical therapy and was referred for advanced 3D running analysis to characterize potential biomechanical contributors and inform rehabilitation and return-to-running progression.

The analysis identified multimodal interlimb asymmetries across kinematic, kinetic, and temporal-spatial measures. Specifically, the symptomatic left limb demonstrated increased knee flexion and reduced ankle plantarflexion during midstance, accompanied by elevated knee joint moments and greater forefoot loading (Figure 5A,C). Together, these findings were consistent with altered load distribution through the symptomatic limb. Temporal-spatial analysis further revealed prolonged stance duration on the left (Figure 5B), indicating greater time under load on the symptomatic extremity during each gait cycle. Ground reaction force data corroborated these findings, with higher peak vertical forces and increased braking impulse on the left side, consistent with disproportionate deceleration loading during running (Figure 6).

Surface EMG analysis provided additional insight into the neuromuscular context of these asymmetries. The left tibialis anterior and medial gastrocnemius demonstrated earlier activation, while the rectus femoris and semitendinosus exhibited delayed recruitment (Figure 7). When interpreted alongside the kinematic and kinetic findings, these activation differences supported a multimodal pattern of altered neuromuscular control and load distribution in the symptomatic limb.

Clinically, the multimodal assessment provided information beyond symptom localization and structural imaging by identifying how the athlete redistributed mechanical and neuromuscular demand during running. These findings informed individualized rehabilitation emphasizing gait retraining, neuromuscular re-education, and restoration of more symmetric load sharing. This case illustrates the translational application of the study framework: synchronized 3D kinematics, kinetics, and sEMG can characterize clinically relevant compensatory strategies that may not be apparent from symptoms, imaging, or observational gait assessment alone.

## 5. Conclusions

Integrated markerless 3D motion analysis, instrumented treadmill kinetics, and sEMG demonstrated that runners with RRIs use clinically distinct, symptom-region-associated strategies to redistribute mechanical and neuromuscular demand across the kinetic chain. Runners presenting with primary hip- and knee-region RRIs demonstrated patterns consistent with redistribution of selected mechanical demands toward the contralateral limb across midstance and/or propulsion, whereas runners with a primary foot/ankle-region RRI combined contralateral kinetic loading with greater symptomatic-limb tibialis anterior demand; the spine-region RRI cohort showed a distinct distal kinematic and gastrocnemius pattern. These findings move clinical interpretation beyond the binary question of whether asymmetry is present toward the more actionable question of where and how the runner compensates. Multimodal biomechanical profiling may therefore provide a functional complement to examination and imaging, support individualized rehabilitation targets, and enable longitudinal assessment of load-sharing recovery. Prospective studies are required before these measures are used for injury prediction or return-to-running clearance.

## Figures and Tables

**Figure 1:**
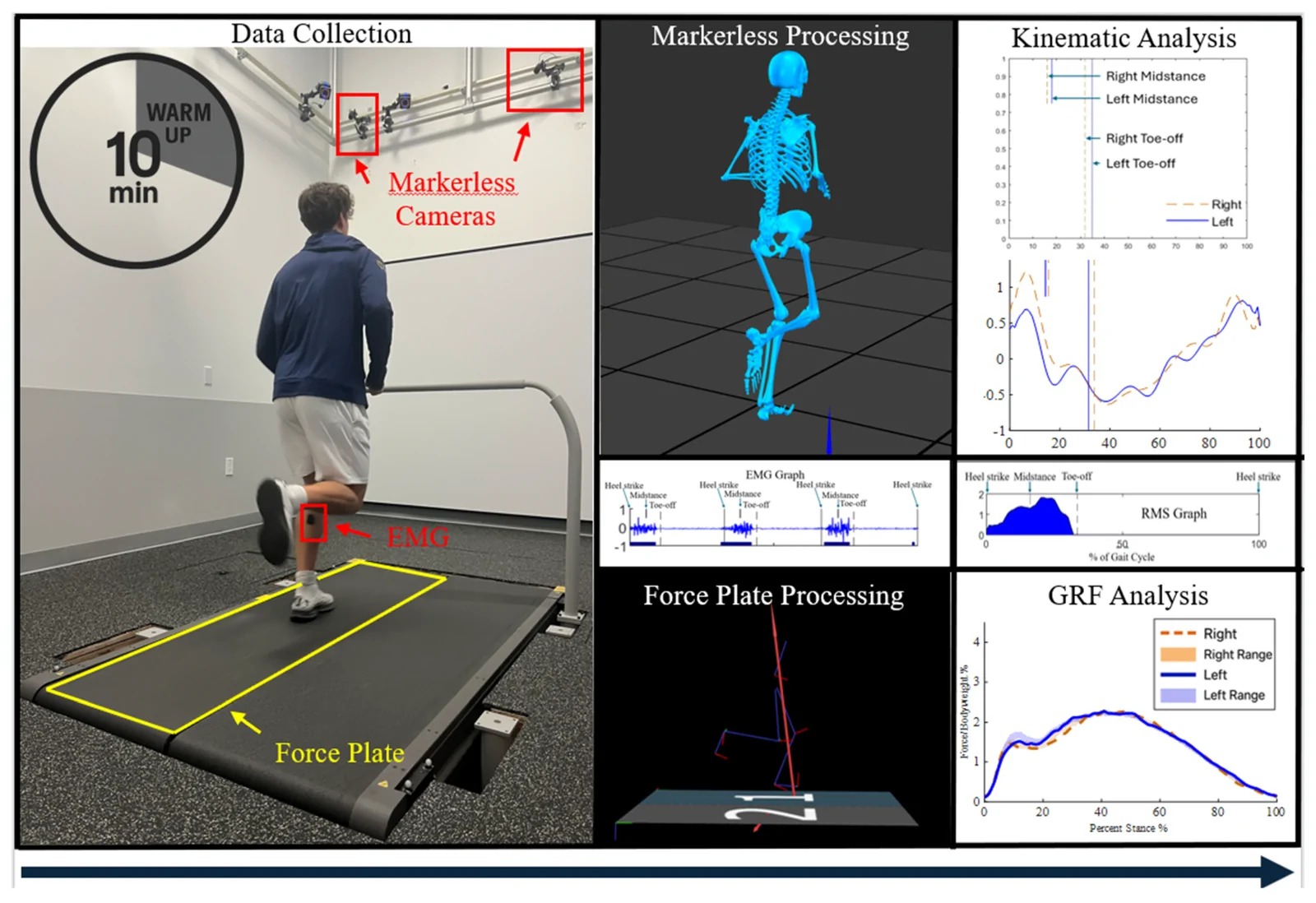
**Integrated 3D running biomechanics platform and data acquisition workflow.** Schematic representation of the multimodal running assessment system. Participants performed steady-state treadmill running on a dual-belt instrumented treadmill with embedded force plates (outlined in yellow). Whole-body kinematics were captured using a markerless motion-capture system, and neuromuscular activity was recorded bilaterally using wireless surface electromyography (sEMG) sensors (highlighted in red). Markerless camera data were processed to generate three-dimensional skeletal reconstructions; sEMG signals were band-pass filtered, root mean square (RMS)-processed, and normalized to maximum voluntary isometric contraction (MVC); force plate signals were filtered and used to compute ground reaction force (GRF)-derived kinetic variables. All data streams were time-synchronized to permit integrated analysis of kinematic, kinetic, and neuromuscular variables across the gait cycle.

**Figure 2:**
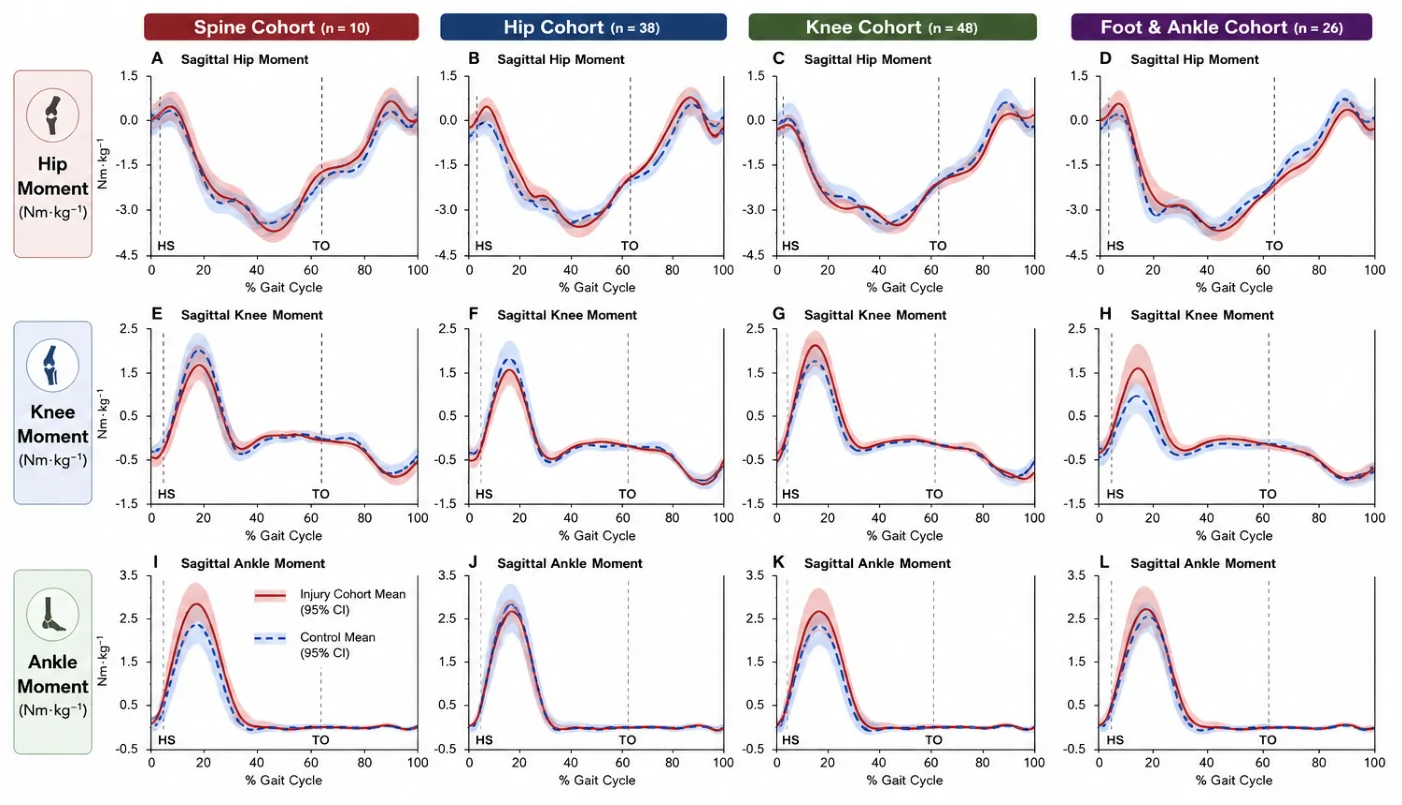
**Sagittal-plane lower-extremity joint moment profiles across RRI cohorts and controls.** Group mean sagittal-plane joint moments for hip (**A**–**D**), knee (**E**–**H**), and ankle (**I**–**L**) plotted across the normalized gait cycle (0–100%). Columns represent clinical cohorts grouped by primary anatomical region of symptoms (spine, hip, knee, foot and ankle). Joint moments are normalized to body mass (Nm·kg^−1^). Solid red lines indicate injury cohort means; dashed blue lines indicate control means. Shaded regions represent 95% confidence intervals.

**Figure 3:**
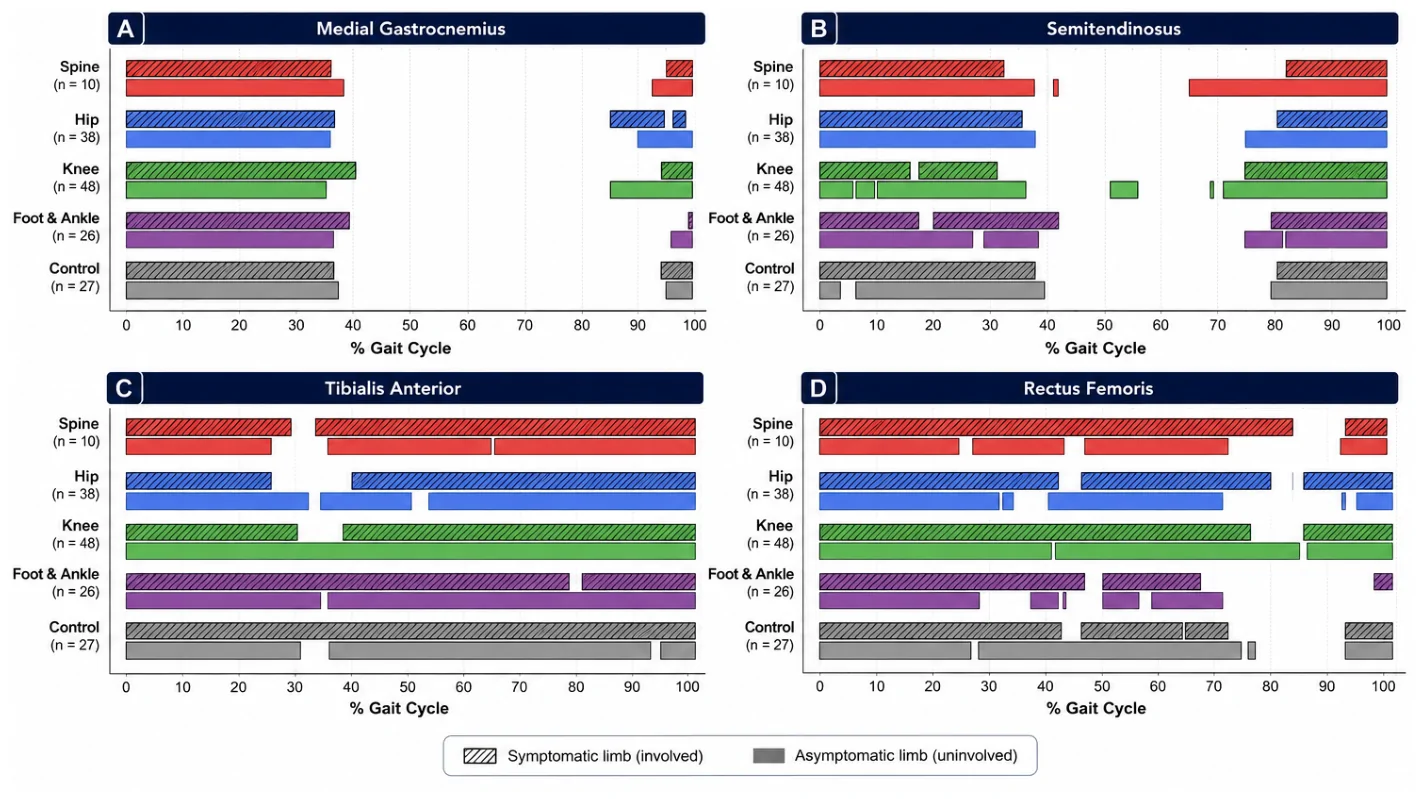
**Lower-Extremity neuromuscular activation timing profiles across RRI cohorts.** Representative normalized sEMG activation profiles for medial gastrocnemius, semitendinosus, tibialis anterior, and rectus femoris across the gait cycle (0–100%). Activation periods are displayed for each RRI cohort (spine, hip, knee, and foot/ankle) and stratified by symptomatic and contralateral limbs for the hip, knee, and foot/ankle cohorts and by dominant and nondominant limbs for the spine cohort. Hatched and solid bars distinguish the two limbs, respectively. Colored bars denote periods during which normalized RMS amplitude exceeded the predefined activation threshold (10% of smoothed peak). Timing is expressed as percentage of the gait cycle.

**Figure 4:**
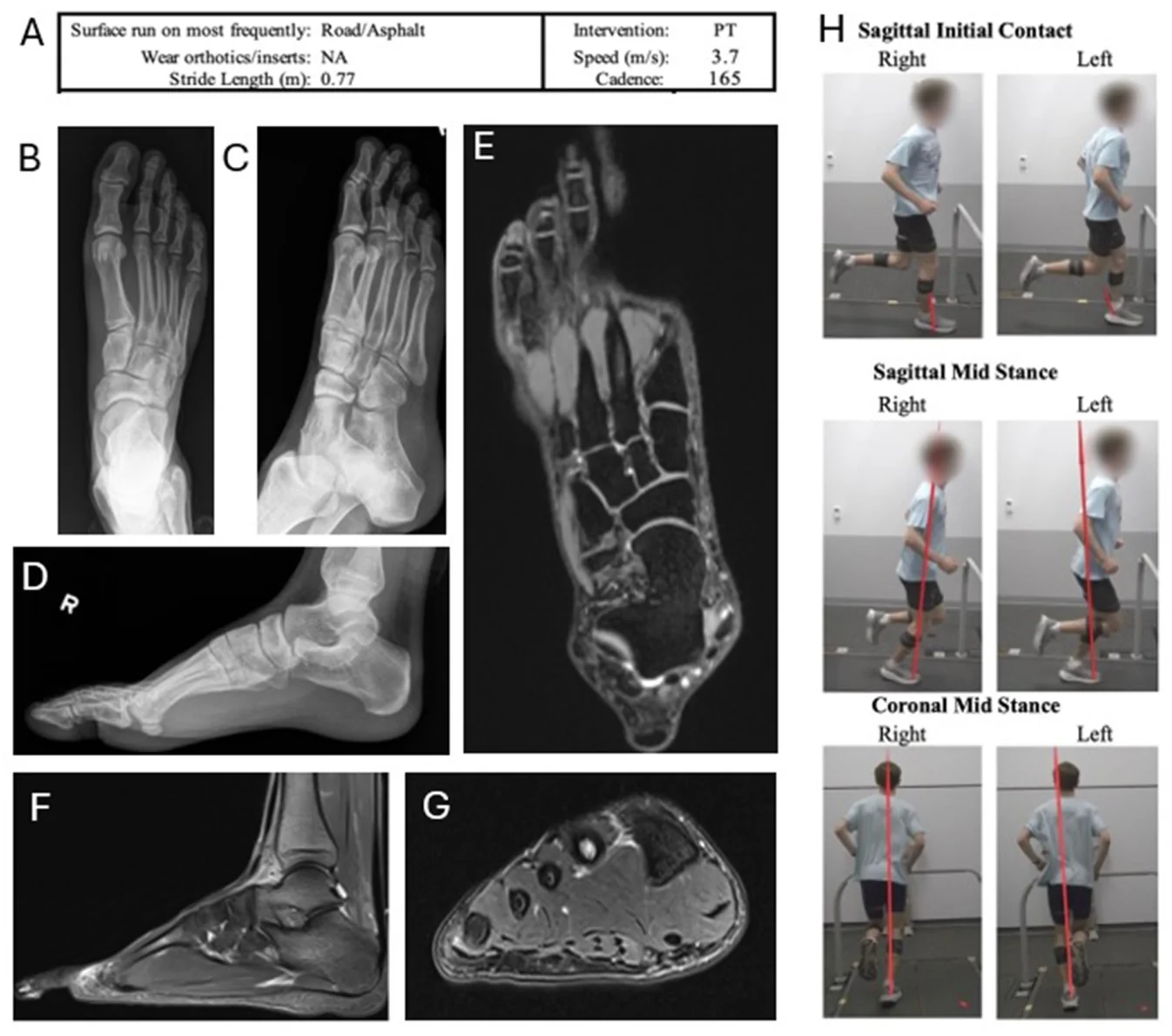
**Clinical imaging and running biomechanics in an athlete with a second metatarsal stress reaction.** (**A**) Athlete profile summarizing habitual running surface, orthotic use, intervention strategy, running speed, cadence, and stride length. NA: Not applicable. (**B**–**D**) Standard weight-bearing radiographs (anteroposterior, oblique, and lateral views) demonstrate no overt osseous abnormality. (**E**–**G**) T2-weighted magnetic resonance imaging (coronal, sagittal, and axial planes) identifying a mid-shaft second metatarsal stress reaction with associated bone marrow oedema. (**H**) Representative snapshots of initial contact and midstance for both limbs; superimposed red vectors indicate phase-specific ground reaction force magnitude and direction. Written informed consent was obtained from the participant for publication of the clinical case and associated images.

**Figure 5:**
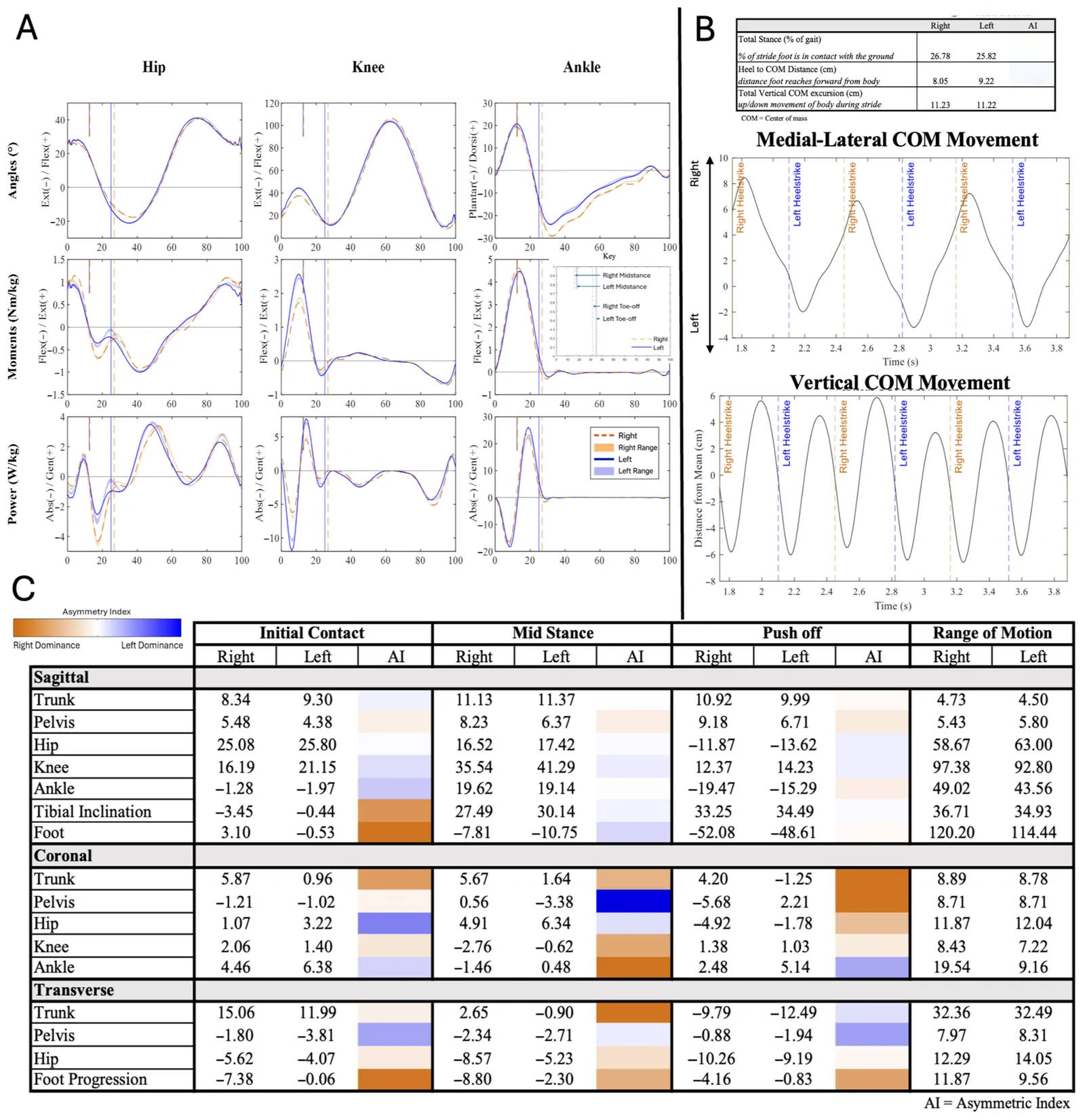
**Lower-extremity kinematic and kinetic profiles in the clinical case illustration.** (**A**) Representative joint angle, joint moment, and joint power trajectories across the gait cycle, with the right limb depicted by an orange dashed line and the left limb by a solid blue line. Vertical markers denote midstance and toe-off events. (**B**) Center-of-mass displacement in the vertical and mediolateral directions, with accompanying temporal-spatial metrics including stance/contact time. (**C**) Mean lower-extremity joint angles at initial contact, midstance, and push-off, along with full-cycle range of motion (ROM). Interlimb asymmetry indices are visualized using a continuous color gradient (orange = right limb; blue = left limb).

**Figure 6:**
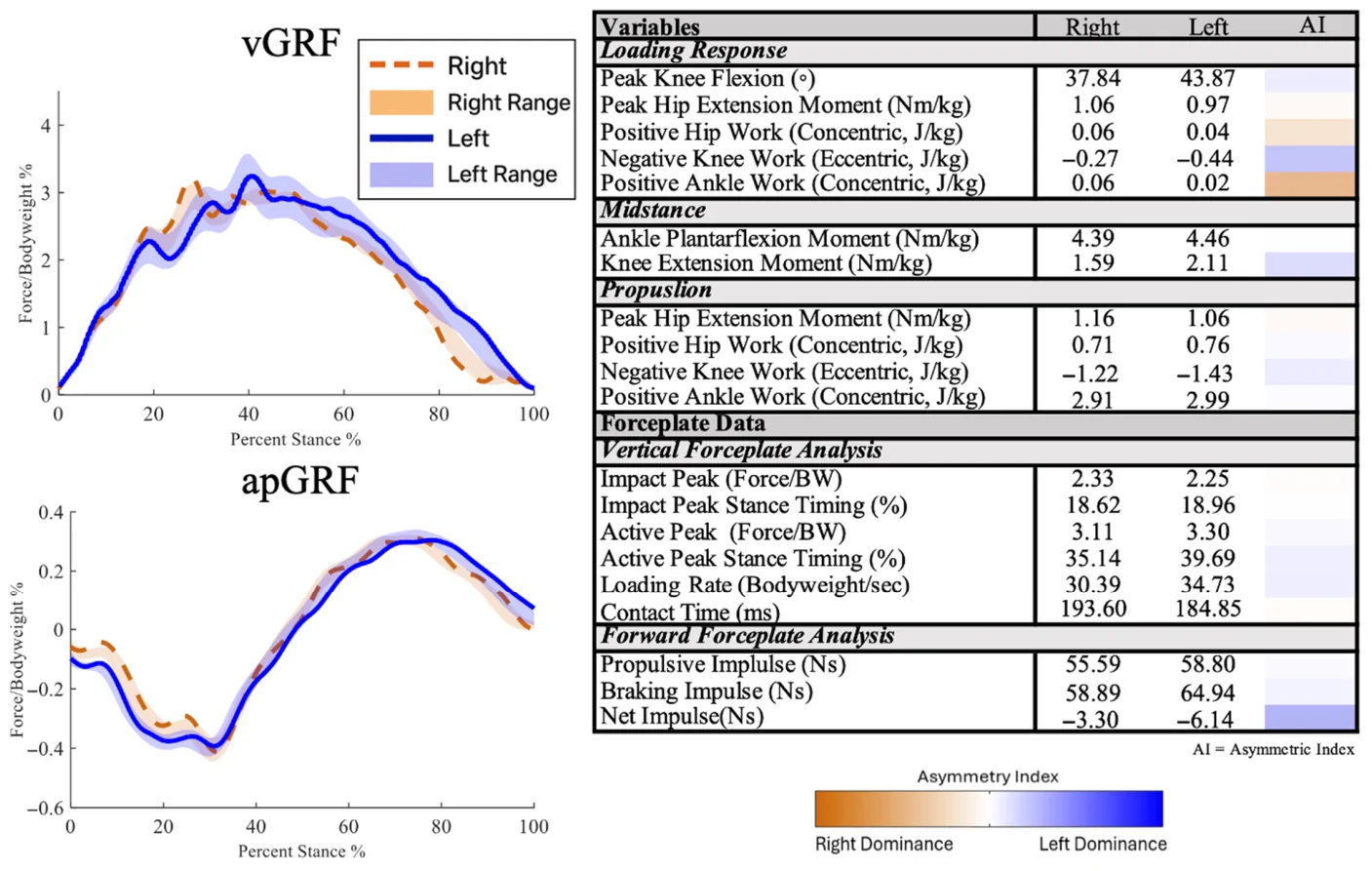
**Ground reaction force and joint kinetic profiles in the clinical case illustration.** Vertical and anterior–posterior ground reaction force (GRF) profiles are presented with 95% confidence intervals (orange, right limb; blue, left limb) across a representative gait cycle. Gait cycle timing is indicated by an orange dashed line (right limb) and a solid blue line (left limb). Summary tables report corresponding lower-extremity joint moments and mechanical work during stance. Inter-limb asymmetry indices for GRF and kinetic measures are illustrated using an orange-to-blue color gradient.

**Figure 7:**
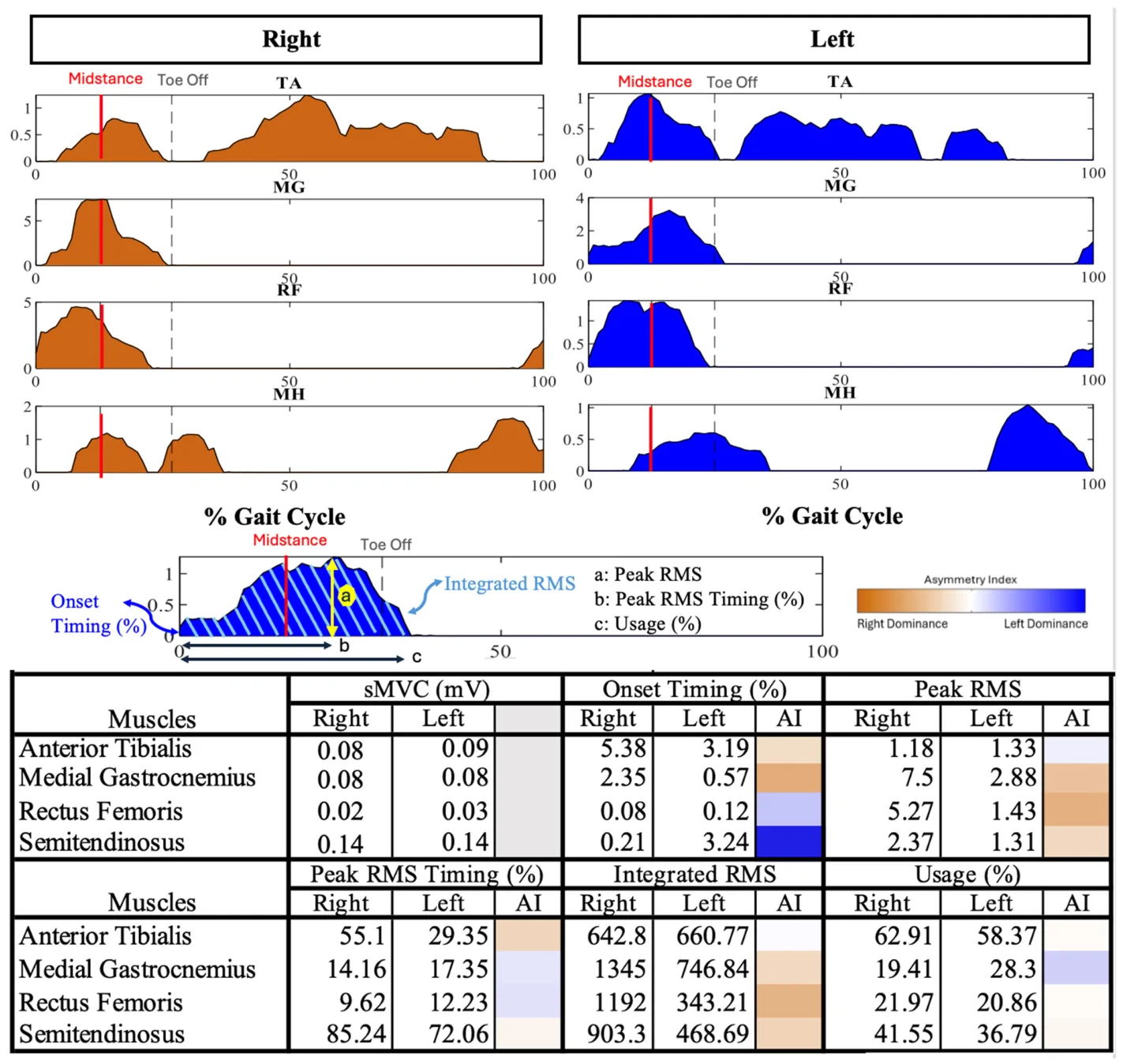
**Neuromuscular activation profiles during running in the clinical case illustration.** Root mean square (RMS) electromyographic activation profiles of the tibialis anterior, medial gastrocnemius, rectus femoris, and semitendinosus are shown across the gait cycle for both limbs. Red vertical lines indicate midstance, and grey dashed lines denote toe-off. Quantified neuromuscular metrics include maximum voluntary contraction (MVC), onset timing (% gait cycle), peak RMS amplitude, timing of peak activation (% gait cycle), integrated RMS, and percentage of muscle activity. Interlimb asymmetry indices are visualized using an orange-to-blue color gradient.

**Table 1:** Participant demographic and running characteristics by RRI cohort and control group.

	Spine	Hip	Knee	Foot & Ankle	Control
Group Size	10	38	48	26	27
Gender (M/F)	3M/7F	16M/21F	25M/23F	17M/9F	14M/13F
Age	37.70 (16.43)	38.08 (17.44)	36.23 (14.61)	37.04 (12.73)	31.41 (15.37)
BMI (kg/m^2^)	23.02 (3.03)	22.42 (3.38)	23.75 (3.61)	24.21 (3.30)	23.00 (3.78)
Height (cm)	169.18 (4.05)	171.62 (7.82)	172.00 (9.80)	176.06 (9.11)	173.38 (11.35)
Weight (kg)	66.12 (10.63)	66.31 (12.72)	70.85 (14.77)	75.19 (12.75)	69.19 (13.08)
Symptomatic/Dominant Side (L/R)	1L/9R	8L/30R	5L/43R	4L/22R	3L/24R
Miles Per Hour (mph)	6.24 (1.20)	5.90 (1.85)	5.94 (1.08)	6.47 (1.08)	7.09 (1.30)
Cadence (steps/min)	164.40 (9.71)	161.95 (10.23)	161.85 (9.40)	158.96 (10.77)	163.74 (11.50)
Stride Length (m)	0.67 (0.09)	0.68 (0.09)	0.65 (0.09)	0.71 (0.08)	0.74 (0.09)
Running Experience					
Beginner	2 (20%)	2 (5%)	5 (10%)	0	0
Intermediate	6 (60%)	19 (50%)	32 (67%)	11 (42%)	6 (22%)
Advanced	0	10 (26%)	8 (17%)	10 (38%)	6 (22%)
Competitive	2 (20%)	7 (18%)	3 (6%)	5 (19%)	15 (56%)

Group-level demographic, anthropometric, and running-related characteristics for runners with spine, hip, knee, or foot/ankle pain and asymptomatic controls. Continuous variables are presented as mean ± standard deviation unless otherwise specified. No statistically significant between-group differences were identified for age, body mass index, height, body mass, cadence, stride length, or self-selected running speed (all *p* > 0.05).

**Table 2:** Principal symptom-region-associated neuromechanical findings during running.

RRI Cohort	Domain/Gait Phase	Principal Finding	Direction of Asymmetry	Model-Based Difference	Adjusted *p*
**Spine**	Neuromuscular	Medial gastrocnemius peak RMS	Dominant > nondominant	0.35	0.037
	Neuromuscular	Medial gastrocnemius integrated RMS	Dominant > nondominant	0.32	0.037
**Hip**	Kinetic—midstance	Knee extension moment	Contralateral > symptomatic	0.14	0.035
	Kinetic—propulsion	Positive hip work	Contralateral > symptomatic	0.27	0.040
	Kinetic—propulsion	Positive ankle work	Contralateral > symptomatic	0.08	0.019
	Kinetic—propulsion	Propulsive impulse	Contralateral > symptomatic	0.07	0.029
	Neuromuscular	Tibialis anterior peak RMS	Symptomatic > contralateral	0.47	0.046
	Neuromuscular	Tibialis anterior integrated RMS	Symptomatic > contralateral	0.38	0.018
**Knee**	Kinetic—loading response	Hip extension moment	Contralateral > symptomatic	0.14	0.013
	Kinetic—propulsion	Propulsive impulse	Contralateral > symptomatic	0.07	0.031
	Kinematic—midstance	Sagittal ankle motion	Interlimb asymmetry	0.14	0.001
	Neuromuscular	Tibialis anterior peak RMS	Contralateral > symptomatic	−0.49	0.023
	Neuromuscular	Semitendinosus onset timing	Delayed on symptomatic limb	0.77	0.035
**Foot/ankle**	Kinetic—midstance	Knee extension moment	Contralateral > symptomatic	0.18	0.017
	Neuromuscular	Tibialis anterior peak RMS	Symptomatic > contralateral	0.52	0.032
	Neuromuscular	Tibialis anterior integrated RMS	Symptomatic > contralateral	0.44	0.013
	Neuromuscular	Tibialis anterior peak timing	Delayed on symptomatic limb	0.34	0.041

## Data Availability

The original contributions presented in this study are included in the article/Supplementary Materials. Further inquiries can be directed to the corresponding author.

## Supplementary Materials

The following supporting information can be downloaded at https://www.mdpi.com/article/10.3390/jfmk11040378/s1, Table S1: Neuromuscular Asymmetry Metrics Across RRI Cohorts and Controls; Table S2: Ground Reaction Force and Kinetic Asymmetry Metrics Across RRI Cohorts and Controls; Table S3: Three-Dimensional Kinematic Asymmetry Metrics Across RRI Cohorts and Controls.
