# Supplementary material for: Symptom-Region-Associated Neuromechanical Compensation During Running: Integrated 3D Kinematic, Kinetic and Electromyographic Profiling of Running-Related Injuries
Source: J Funct Morphol Kinesiol. 2026 Sep 23;11(4):378. doi: 10.3390/jfmk11040378 (PMC13692485; doi:10.3390/jfmk11040378)
Supplement: Supplementary file 1 [file jfmk-11-00378-s001.zip › jfmk-4541231-supplementary.pdf]

Supplementary Table S1. Neuromuscular Asymmetry Metrics Across RRI Cohorts and Controls.

	Tibialis Anterior			Medial Gastrocnemius			Rectus Femoris			Semitendinosus		
	Mean 95CI	Diff 95CI	P Val	Mean 95CI	Diff 95CI	P Val	Mean 95CI	Diff 95CI	P Val	Mean 95CI	Diff 95CI	P Val
**MVC Voltage – Average active phase amplitude**												
Control	0.14 [-0.04, 0.32]			-0.04 [-0.16, 0.08]			-0.13 [-0.33, 0.08]			-0.11 [-0.30, 0.08]		
Spine	0.40 [0.05, 0.75]	0.28 [-0.12, 0.69]	0.159	-0.18 [-0.30, -0.06]	-0.14 [-0.39, 0.10]	0.235	-0.24 [-0.49, 0.01]	-0.10 [-0.52, 0.32]	0.619	-0.12 [-0.51, 0.27]	-0.08 [-0.49, 0.32]	0.682
Hip	-0.34 [-0.63, -0.05]	-0.30 [-0.69, 0.09]	0.128	-0.01 [-0.11, 0.09]	-0.01 [-0.18, 0.16]	0.933	0.08 [-0.08, 0.23]	0.21 [-0.06, 0.49]	0.126	-0.06 [-0.19, 0.07]	0.04 [-0.20, 0.28]	0.737
Knee	0.54 [0.38, 0.71]	0.53 [0.24, 0.83]	0.001 ‡	-0.01 [-0.10, 0.09]	0.04 [-0.14, 0.23]	0.649	-0.12 [-0.24, -0.01]	-0.02 [-0.27, 0.24]	0.906	-0.01 [-0.18, 0.15]	0.05 [-0.26, 0.35]	0.768
Foot & Ankle	-0.23 [-0.54, 0.08]	-0.34 [-0.71, 0.03]	0.071	0.04 [-0.22, 0.30]	0.11 [-0.19, 0.40]	0.468	-0.05 [-0.27, 0.16]	0.07 [-0.24, 0.39]	0.640	-0.06 [-0.20, 0.08]	0.04 [-0.21, 0.30]	0.732
**Onset Timing – First point in the gait cycle that the muscle signal exceeded the activation threshold.**												
Control	-0.37 [-0.73, -0.01]			-0.22 [-0.66, 0.22]			-0.31 [-0.88, 0.25]			-0.32 [-0.84, 0.20]		
Spine	-0.76 [-1.89, 0.36]	-0.39 [-1.36, 0.57]	0.409	0.45 [-0.19, 1.09]	0.57 [-0.33, 1.48]	0.204	0.70 [0.06, 1.33]	1.10 [-0.03, 2.22]	0.056	-1.49 [-3.10, 0.11]	-1.19 [-2.57, 0.19]	0.089
Hip	-0.00 [-0.38, 0.38]	0.38 [-0.22, 0.97]	0.214	0.01 [-0.44, 0.46]	0.17 [-0.55, 0.88]	0.642	0.07 [-0.52, 0.66]	0.36 [-0.56, 1.28]	0.433	0.39 [-0.19, 0.96]	0.76 [-0.11, 1.63]	0.085
Knee	-0.62	-0.39	0.288	0.18	0.40	0.427	0.23	0.52	0.185	0.45	0.77	0.035 *

	[-1.06, -0.19]	[-1.13, 0.34]		[-0.40, 0.77]	[-0.60, 1.39]		[-0.16, 0.62]	[-0.26, 1.30]		[0.10, 0.79]	[0.06, 1.47]	
Foot & Ankle	0.02 [-0.38, 0.41]	0.35 [-0.21, 0.92]	0.216	-0.21 [-0.86, 0.45]	-0.19 [-0.99, 0.60]	0.626	0.19 [-0.71, 1.09]	0.56 [-0.53, 1.66]	0.305	0.01 [-0.93, 0.95]	0.42 [-0.68, 1.52]	0.445
**Peak RMS – Maximum RMS magnitude during the gait cycle**												
Control	-0.31 [-0.60, -0.02]			-0.09 [-0.25, 0.07]			0.03 [-0.22, 0.29]			-0.00 [-0.24, 0.24]		
Spine	-0.35 [-0.72, 0.02]	-0.05 [-0.64, 0.54]	0.869	0.28 [0.08, 0.47]	0.35 [0.02, 0.67]	0.037 *	0.16 [-0.03, 0.35]	0.13 [-0.37, 0.63]	0.591	-0.20 [-0.74, 0.33]	-0.12 [-0.65, 0.42]	0.659
Hip	0.26 [-0.04, 0.56]	0.47 [0.01, 0.94]	0.046 *	0.06 [-0.12, 0.24]	0.19 [-0.09, 0.47]	0.190	0.03 [-0.20, 0.25]	-0.05 [-0.43, 0.32]	0.773	-0.04 [-0.18, 0.11]	-0.01 [-0.30, 0.28]	0.958
Knee	-0.64 [-0.87, -0.42]	-0.49 [-0.91, -0.07]	0.023 *	0.09 [-0.02, 0.19]	0.14 [-0.07, 0.36]	0.191	0.09 [-0.09, 0.27]	0.09 [-0.27, 0.44]	0.619	0.01 [-0.21, 0.23]	-0.01 [-0.40, 0.39]	0.975
Foot & Ankle	0.20 [-0.14, 0.55]	0.52 [0.05, 1.00]	0.032 *	-0.23 [-0.51, 0.04]	-0.23 [-0.55, 0.08]	0.138	0.06 [-0.24, 0.36]	0.05 [-0.36, 0.46]	0.807	-0.14 [-0.45, 0.17]	-0.12 [-0.53, 0.30]	0.572
**Peak RMS Timing – Percentage of the gait cycle at which peak RMS occurred**												
Control	0.11 [-0.14, 0.36]			-0.03 [-0.10, 0.05]			0.08 [-0.07, 0.24]			0.08 [0.00, 0.15]		
Spine	0.36 [0.02, 0.69]	0.32 [-0.17, 0.81]	0.195	0.05 [-0.06, 0.15]	0.05 [-0.10, 0.21]	0.492	-0.02 [-0.28, 0.24]	-0.11 [-0.44, 0.22]	0.497	0.01 [-0.07, 0.08]	-0.06 [-0.22, 0.10]	0.429
Hip	-0.04 [-0.38, 0.30]	-0.14 [-0.58, 0.30]	0.531	-0.03 [-0.07, 0.02]	0.02 [-0.08, 0.11]	0.701	-0.07 [-0.22, 0.09]	-0.19 [-0.44, 0.05]	0.111	-0.01 [-0.06, 0.05]	-0.08 [-0.18, 0.03]	0.142
Knee	-0.14	-0.06	0.727	0.05	0.06	0.339	-0.07	-0.21	0.048 *	-0.10	-0.15	0.046 *

	[-0.36, 0.09]	[-0.40, 0.28]		[-0.02, 0.12]	[-0.07, 0.19]		[-0.18, 0.04]	[-0.43, -0.00]		[-0.19, -0.01]	[-0.30, -0.00]	
Foot & Ankle	0.42 [0.26, 0.58]	0.34 [0.01, 0.67]	0.041 *	0.08 [0.01, 0.15]	0.10 [-0.02, 0.21]	0.096	0.04 [-0.15, 0.24]	-0.06 [-0.31, 0.20]	0.658	0.06 [-0.09, 0.21]	-0.00 [-0.17, 0.17]	0.995
**Integrated RMS – Total muscle activity**												
Control	-0.20 [-0.42, 0.02]			-0.08 [-0.23, 0.07]			0.14 [-0.10, 0.38]			0.03 [-0.20, 0.26]		
Spine	-0.09 [-0.43, 0.24]	0.11 [-0.35, 0.57]	0.619	0.26 [0.09, 0.44]	0.32 [0.02, 0.62]	0.037 *	0.04 [-0.30, 0.39]	-0.11 [-0.61, 0.39]	0.658	-0.16 [-0.71, 0.39]	-0.12 [-0.64, 0.41]	0.656
Hip	0.27 [0.07, 0.46]	0.38 [0.07, 0.70]	0.018 *	-0.00 [-0.20, 0.19]	0.12 [-0.17, 0.40]	0.417	-0.02 [-0.28, 0.24]	-0.21 [-0.61, 0.19]	0.304	-0.06 [-0.22, 0.10]	-0.10 [-0.39, 0.19]	0.502
Knee	-0.45 [-0.63, -0.27]	-0.33 [-0.66, 0.01]	0.055	0.10 [-0.02, 0.22]	0.13 [-0.10, 0.37]	0.251	0.08 [-0.06, 0.23]	-0.03 [-0.35, 0.28]	0.824	-0.01 [-0.22, 0.21]	-0.01 [-0.40, 0.37]	0.952
Foot & Ankle	0.24 [0.01, 0.47]	0.44 [0.10, 0.77]	0.013 *	-0.15 [-0.40, 0.10]	-0.15 [-0.44, 0.13]	0.285	0.04 [-0.27, 0.34]	-0.09 [-0.50, 0.31]	0.640	-0.09 [-0.34, 0.17]	-0.12 [-0.49, 0.24]	0.491
**Usage -- Percentage of the gait cycle that the muscle was active**												
Control	0.11 [-0.05, 0.27]			0.03 [-0.06, 0.12]			0.10 [-0.07, 0.26]			0.00 [-0.13, 0.13]		
Spine	0.39 [-0.21, 0.98]	0.29 [-0.18, 0.76]	0.215	0.00 [-0.12, 0.13]	-0.05 [-0.23, 0.12]	0.541	-0.25 [-0.60, 0.10]	-0.35 [-0.74, 0.04]	0.075	0.07 [-0.16, 0.29]	0.09 [-0.20, 0.37]	0.529
Hip	-0.02 [-0.19, 0.15]	-0.12 [-0.39, 0.15]	0.376	-0.02 [-0.10, 0.06]	-0.05 [-0.18, 0.08]	0.448	-0.01 [-0.17, 0.16]	-0.11 [-0.38, 0.15]	0.393	-0.02 [-0.14, 0.09]	-0.02 [-0.21, 0.17]	0.801
Knee	0.24	0.24	0.055	-0.00	-0.10	0.149	-0.33	-0.42	0.509	0.03	0.06	0.596

	[0.11, 0.37]	[-0.00, 0.48]		[-0.08, 0.07]	[-0.24, 0.04]		[-1.16, 0.51]	[-1.70, 0.85]		[-0.10, 0.15]	[-0.16, 0.28]	
Foot & Ankle	-0.00 [-0.20, 0.20]	-0.13 [-0.40, 0.14]	0.335	0.05 [-0.02, 0.12]	0.01 [-0.11, 0.14]	0.802	0.01 [-0.27, 0.28]	-0.09 [-0.42, 0.24]	0.588	0.01 [-0.21, 0.22]	-0.01 [-0.27, 0.25]	0.948

Surface electromyography (sEMG)–derived variables including peak RMS amplitude, integrated RMS, onset timing, peak timing, and muscle activation duration for runners with spine, hip, knee, or foot/ankle pathology and asymptomatic controls. EMG amplitudes were normalized to maximum voluntary isometric contraction (MVC). Asymmetry index (AI) was calculated as log(Symptomatic / Asymptomatic). AI = 0 indicates symmetry; positive values indicate greater magnitude on the symptomatic limb; negative values indicate greater magnitude on the contralateral limb.

Supplementary Table S2. Ground Reaction Force and Kinetic Asymmetry Metrics Across RRI Cohorts and Controls.

	Control	Spine			Hip			Knee			Foot and Ankle		
	**Mean [95CI]**	**Mean [95CI]**	**Diff [95CI]**	**P Val**	**Mean [95CI]**	**Diff [95CI]**	**P Val**	**Mean [95CI]**	**Diff [95CI]**	**P Val**	**Mean [95CI]**	**Diff [95CI]**	**P Val**
Stance Percentage and COM Dynamics													
Total Stance	-0.01 [-0.01, 0.00]	0.01 [-0.00, 0.03]	0.02 [0.01,0.04]	0.010 *	0.01 [-0.00, 0.02]	0.02 [0.00, 0.03]	0.050 *	0.01 [-0.00, 0.02]	0.02 [0.00, 0.03]	0.050 *	0.01 [-0.00, 0.02]	0.02 [0.00, 0.03]	0.028 *
Heel to COM Distance	0.03 [-0.02, 0.07]	0.04 [-0.01, 0.09]	-0.01 [-0.09,0.07]	0.858	0.01 [-0.04, 0.06]	-0.02 [-0.10, 0.06]	0.656	0.03 [-0.03, 0.08]	0.00 [-0.08, 0.09]	0.913	0.00 [-0.05, 0.06]	-0.04 [-0.11,0.04]	0.310
Vertical COM Excursion	0.00 [0.00, 0.00]	0.00 [-0.00, 0.00]	-0.00 [-0.00, 0.00]	0.530	0.00 [-0.00, 0.00]	-0.00 [-0.00, 0.00]	0.118	-0.00 [-0.00, 0.00]	-0.00 [-0.00, 0.00]	0.291	0.04 [-0.01, 0.09]	-0.01 [-0.09, 0.07]	0.858
Loading Response													
Peak Knee Flexion	-0.01 [-0.04, 0.02]	-0.04 [-0.10, 0.01]	-0.02 [-0.09, 0.04]	0.446	0.01 [-0.02, 0.04]	0.03 [-0.01, 0.08]	0.160	0.00 [-0.02, 0.03]	-0.00 [-0.05, 0.04]	0.882	0.01 [-0.02, 0.04]	0.02 [-0.03, 0.07]	0.392
Peak Hip Extension Moment	-0.06 [-0.14, 0.01]	-0.01 [-0.10, 0.08]	0.04 [-0.11, 0.19]	0.598	-0.04 [-0.09, 0.01]	0.03 [-0.07, 0.12]	0.596	0.05 [-0.01, 0.12]	0.14 [0.03, 0.26]	0.013 *	0.03 [-0.06, 0.12]	0.08 [-0.04, 0.21]	0.195
Positive Hip Work	-0.41 [-1.05, 0.23]	-0.85 [-2.12, 0.42]	-0.40 [-1.83, 1.03]	0.574	-0.07 [-0.55, 0.41]	0.17 [-0.67, 1.02]	0.682	-0.34 [-0.78, 0.10]	-0.12 [-0.98, 0.73]	0.775	-0.52 [-1.19, 0.15]	-0.19 [-1.18, 0.80]	0.699
Negative Knee Work	-0.05 [-0.16, 0.05]	-0.12 [-0.34, 0.11]	-0.00 [-0.23, 0.23]	0.985	0.04 [-0.07, 0.15]	0.17 [-0.00, 0.33]	0.052	0.07 [-0.12, 0.25]	0.04 [-0.25, 0.33]	0.780	0.06 [-0.08, 0.21]	0.13 [-0.06, 0.32]	0.170
Positive Ankle Work	-1.00 [-2.26, 0.25]	-0.86 [-1.40, -0.33]	-0.14 [-2.43, 2.15]	0.902	0.16 [-0.61, 0.93]	1.03 [-0.50, 2.56]	0.183	-0.83 [-1.36, -0.30]	-0.25 [-1.56, 1.07]	0.706	0.08 [-0.93, 1.08]	0.82 [-0.89, 2.52]	0.342
Midstance													

Ankle Plantarflexion Moment	-0.01 [-0.03, 0.01]	-0.04 [-0.06, -0.01]	-0.02 [-0.06, 0.02]	0.368	0.00 [-0.03, 0.03]	0.02 [-0.03, 0.06]	0.457	-0.01 [-0.03, 0.00]	-0.01 [-0.04, 0.03]	0.727	-0.03 [-0.08, 0.01]	-0.02 [-0.07, 0.03]	0.481
Knee Extension Moment	-0.08 [-0.20, 0.04]	-0.08 [-0.25, 0.09]	0.09 [-0.13, 0.30]	0.403	0.03 [-0.04, 0.09]	0.14 [0.01, 0.28]	0.035 *	0.03 [-0.06, 0.13]	0.11 [-0.06, 0.28]	0.209	0.07 [-0.01, 0.15]	0.18 [0.03, 0.33]	0.017 *
Propuslion												
Peak Hip Extension Moment	0.01 [-0.03, 0.04]	-0.01 [-0.06, 0.05]	-0.01 [-0.09, 0.07]	0.755	0.00 [-0.02, 0.03]	-0.00 [-0.05, 0.05]	0.958	0.03 [0.00, 0.06]	0.03 [-0.03, 0.08]	0.333	0.01 [-0.04, 0.05]	-0.00 [-0.06, 0.06]	0.904
Positive Hip Work	-0.12 [-0.29, 0.05]	-0.14 [-0.36, 0.08]	-0.02 [-0.36, 0.33]	0.916	0.16 [0.01, 0.32]	0.27 [0.01, 0.53]	0.040 *	-0.01 [-0.12, 0.09]	0.08 [-0.14, 0.30]	0.456	-0.00 [-0.15, 0.14]	0.13 [-0.12, 0.37]	0.302
Negative Knee Work	-0.02 [-0.06, 0.02]	-0.09 [-0.16, -0.01]	-0.07 [-0.16, 0.02]	0.129	0.01 [-0.03, 0.05]	0.05 [-0.01, 0.12]	0.092	0.00 [-0.05, 0.06]	-0.00 [-0.09, 0.08]	0.912	0.03 [-0.05, 0.11]	0.06 [-0.03, 0.15]	0.182
Positive Ankle Work	-0.06 [-0.11, -0.02]	-0.09 [-0.18, -0.00]	-0.02 [-0.12, 0.08]	0.696	0.02 [-0.02, 0.06]	0.08 [0.01, 0.15]	0.019 *	-0.05 [-0.10, -0.00]	0.01 [-0.07, 0.09]	0.764	-0.01 [-0.07, 0.05]	0.05 [-0.02, 0.13]	0.174
Vertical Forceplate Analysis												
Impact Peak	-0.01 [-0.04, 0.02]	-0.01 [-0.05, 0.03]	0.00 [-0.06, 0.06]	0.963	0.02 [-0.01, 0.05]	0.04 [-0.00, 0.08]	0.080	-0.01 [-0.04, 0.02]	0.01 [-0.05, 0.07]	0.800	0.00 [-0.04, 0.04]	0.01 [-0.04, 0.07]	0.604
Impact Peak Stance Timing	0.01 [-0.03, 0.04]	0.10 [0.02, 0.17]	0.06 [-0.01, 0.13]	0.102	-0.04 [-0.09, 0.02]	-0.07 [-0.15, 0.00]	0.063	0.00 [-0.04, 0.04]	-0.03 [-0.10, 0.05]	0.493	0.06 [-0.02, 0.14]	0.04 [-0.05, 0.14]	0.337
Active Peak	0.00 [-0.01, 0.02]	-0.02 [-0.04, -0.01]	-0.02 [-0.04, 0.01]	0.276	0.01 [-0.01, 0.02]	0.01 [-0.02, 0.03]	0.572	-0.01 [-0.02, 0.01]	-0.01 [-0.03, 0.02]	0.644	-0.00 [-0.02, 0.02]	-0.00 [-0.03, 0.02]	0.967
Active Peak Stance Timing	-0.01 [-0.04, 0.02]	0.02 [-0.05, 0.10]	0.03 [-0.04, 0.11]	0.365	0.01 [-0.03, 0.04]	0.02 [-0.03, 0.07]	0.474	-0.00 [-0.02, 0.02]	0.02 [-0.02, 0.06]	0.388	0.02 [-0.03, 0.06]	0.03 [-0.03, 0.09]	0.268

Loading Rate	-0.01 [-0.08, 0.06]	-0.15 [-0.28, -0.03]	-0.13 [-0.28, 0.02]	0.080	0.09 [-0.00, 0.19]	0.13 [-0.01, 0.27]	0.072	-0.07 [-0.13, -0.01]	-0.04 [-0.15, 0.07]	0.504	-0.07 [-0.18, 0.03]	-0.07 [-0.20, 0.07]	0.326
Contact Time	-0.00 [-0.04, 0.03]	0.01 [-0.00, 0.03]	0.01 [-0.05, 0.07]	0.801	-0.00 [-0.01, 0.01]	0.00 [-0.03, 0.04]	0.862	0.01 [0.00, 0.02]	0.01 [-0.03, 0.04]	0.700	0.01 [-0.01, 0.02]	0.00 [-0.04, 0.04]	0.935
Forward Forceplate Analysis													
Propulsive Implulse	-0.05 [-0.08, -0.01]	-0.02 [-0.08, 0.04]	0.04 [-0.03, 0.12]	0.234	0.01 [-0.02, 0.05]	0.07 [0.01, 0.13]	0.029 *	0.01 [-0.03, 0.04]	0.07 [0.01, 0.13]	0.031 *	-0.02 [-0.08, 0.04]	0.04 [-0.03, 0.11]	0.300
Braking Impulse	0.01 [-0.03, 0.05]	0.02 [-0.07, 0.11]	0.01 [-0.09, 0.10]	0.888	-0.01 [-0.09, 0.06]	0.01 [-0.09, 0.11]	0.864	0.03 [-0.03, 0.08]	0.02 [-0.07, 0.10]	0.708	-0.02 [-0.08, 0.03]	-0.03 [-0.11, 0.04]	0.363
Net Impulse	0.19 [-0.30, 0.67]	-0.38 [-1.18, 0.42]	-0.62 [-1.65, 0.40]	0.226	0.64 [0.16, 1.11]	0.21 [-0.54, 0.95]	0.581	-0.15 [-0.63, 0.32]	-0.43 [-1.26, 0.40]	0.305	0.37 [-0.50, 1.24]	0.13 [-0.92, 1.19]	0.803

Vertical and anterior–posterior ground reaction force–derived kinetic variables including stance time, loading rate, impact peak, activation peak, vertical impulse, braking impulse, propulsive impulse, and net impulse. All forces were normalized to body weight; joint moments were normalized to body mass ($Nm \cdot kg^{-1}$). Asymmetry index (AI) was calculated as log(Symptomatic / Asymptomatic). AI = 0 indicates symmetry; positive values indicate greater loading on the symptomatic limb; negative values indicate greater loading on the contralateral limb.

Supplementary Table S3. Three-Dimensional Kinematic Asymmetry Metrics Across RRI Cohorts and Controls.

	Initial Contact			Midstance			Toe Off			Range of Motion		
	Mean 95CI	Diff 95CI	P Val	Mean 95CI	Diff 95CI	P Val	Mean 95CI	Diff 95CI	P Val	Mean 95CI	Diff 95CI	P Val
**Control**												
**Sagittal**												
Trunk	0.23 [-0.30, 0.76]			0.19 [-0.22, 0.60]			0.34 [-0.25, 0.93]			-0.00 [-0.01, 0.00]		
Pelvis	0.48 [-0.19, 1.15]			0.66 [0.06, 1.26]			-0.07 [-0.48, 0.34]			0.00 [-0.00, 0.00]		
Hip	0.01 [-0.02, 0.05]			-0.05 [-0.13, 0.02]			-0.04 [-0.09, 0.01]			-0.00 [-0.02, 0.01]		
Knee	0.04 [-0.03, 0.10]			-0.02 [-0.05, 0.01]			-0.01 [-0.10, 0.09]			-0.01 [-0.03, 0.01]		
Ankle	-0.24 [-0.54, 0.06]			-0.04 [-0.09, 0.01]			-0.06 [-0.16, 0.04]			-0.01 [-0.05, 0.02]		
Tibial Inclination	-0.24 [-0.66, 0.19]			-0.01 [-0.04, 0.01]			-0.01 [-0.02, 0.01]			-0.01 [-0.03, 0.01]		
Foot	-0.11 [-0.40, 0.19]			0.01 [-0.15, 0.16]			-0.01 [-0.03, 0.01]			-0.00 [-0.01, 0.01]		
**Coronal**												
Trunk	-0.08 [-0.15, 0.00]			0.01 [-0.02, 0.05]			0.04 [-0.02, 0.11]			0.02 [-0.00, 0.03]		

Pelvis	0.00 [-0.05, 0.05]			-0.01 [-0.05, 0.03]			0.01 [-0.02, 0.04]			-0.01 [-0.02, 0.01]		
Hip	0.55 [-0.05, 1.15]			0.05 [-0.28, 0.38]			0.11 [-0.37, 0.58]			0.01 [-0.04, 0.07]		
Knee	-0.31 [-0.88, 0.26]			-0.22 [-0.64, 0.19]			-0.28 [-0.87, 0.31]			-0.03 [-0.16, 0.10]		
Ankle	0.24 [0.01, 0.47]			-0.06 [-0.63, 0.51]			-0.03 [-0.28, 0.21]			-0.02 [-0.15, 0.12]		
**Transverse**												
Trunk	0.05 [-0.06, 0.17]			-0.17 [-0.68, 0.33]			-0.06 [-0.17, 0.04]			-0.00 [-0.00, -0.00]		
Pelvis	-0.26 [-0.69, 0.16]			0.17 [-0.30, 0.64]			0.05 [-0.33, 0.43]			-0.00 [-0.01, 0.00]		
Hip	0.06 [-0.34, 0.47]			-0.20 [-0.56, 0.17]			-0.32 [-0.68, 0.04]			-0.04 [-0.15, 0.07]		
Foot Progression	0.26 [-0.27, 0.78]			0.07 [-0.25, 0.39]			-0.07 [-0.48, 0.34]			0.04 [-0.09, 0.16]		
**Spine**												
**Sagittal**												
Trunk	-0.37 [-1.46, 0.72]	-0.80 [-1.97, 0.38]	0.177	0.11 [-1.16, 1.38]	-0.48 [-1.49, 0.53]	0.338	0.24 [-0.75, 1.23]	-0.04 [-1.29, 1.22]	0.955	-0.00 [-0.01, 0.01]	0.01 [-0.00, 0.02]	0.219

Pelvis	-0.30 [-1.52, 0.93]	-0.86 [-2.32, 0.60]	0.242	1.32 [0.07, 2.56]	0.56 [-0.80, 1.92]	0.408	-0.97 [-1.52, -0.42]	-1.02 [-1.84, -0.20]	0.016 *	-0.00 [-0.01, 0.01]	-0.00 [-0.01, 0.01]	0.694
Hip	0.02 [-0.04, 0.08]	-0.00 [-0.07, 0.07]	0.919	-0.08 [-0.19, 0.02]	0.02 [-0.13, 0.16]	0.821	-0.10 [-0.21, 0.01]	-0.03 [-0.15, 0.08]	0.590	-0.01 [-0.06, 0.05]	-0.01 [-0.06, 0.04]	0.755
Knee	-0.06 [-0.15, 0.03]	-0.10 [-0.23, 0.04]	0.156	-0.06 [-0.13, 0.01]	-0.02 [-0.09, 0.05]	0.634	0.14 [-0.03, 0.31]	0.15 [-0.05, 0.36]	0.140	-0.01 [-0.05, 0.04]	0.01 [-0.03, 0.05]	0.732
Ankle	0.26 [-0.39, 0.91]	0.51 [-0.19, 1.21]	0.145	0.11 [0.04, 0.19]	0.16 [0.06, 0.26]	0.003 †	-0.24 [-0.34, -0.14]	-0.13 [-0.31, 0.05]	0.141	0.02 [-0.05, 0.08]	0.02 [-0.05, 0.10]	0.547
Tibial Inclination	0.24 [-0.15, 0.63]	0.31 [-0.49, 1.10]	0.438	-0.04 [-0.09, 0.01]	-0.01 [-0.06, 0.04]	0.630	0.02 [0.00, 0.05]	0.04 [0.00, 0.07]	0.033 *	0.05 [0.02, 0.07]	0.06 [0.02, 0.09]	0.002 †
Foot	-0.02 [-0.47, 0.44]	0.05 [-0.56, 0.67]	0.860	-0.60 [-0.92, -0.29]	-0.51 [-0.84, -0.19]	0.003 †	-0.04 [-0.07, -0.00]	-0.01 [-0.05, 0.03]	0.524	-0.03 [-0.06, 0.00]	-0.03 [-0.06, -0.00]	0.026 *
**Coronal**												
Trunk	-0.06 [-0.15, 0.04]	0.02 [-0.14, 0.17]	0.807	-0.03 [-0.08, 0.02]	-0.04 [-0.11, 0.04]	0.355	0.03 [-0.07, 0.13]	-0.01 [-0.14, 0.12]	0.884	0.02 [-0.00, 0.04]	0.00 [-0.03, 0.03]	0.992
Pelvis	0.04 [-0.15, 0.22]	0.02 [-0.13, 0.16]	0.819	-0.07 [-0.16, 0.02]	-0.06 [-0.15, 0.03]	0.170	0.01 [-0.19, 0.21]	0.02 [-0.12, 0.16]	0.773	-0.01 [-0.03, 0.01]	-0.01 [-0.03, 0.02]	0.552
Hip	1.04 [0.59, 1.50]	0.54 [-0.59, 1.67]	0.338	0.46 [0.08, 0.84]	0.47 [-0.17, 1.12]	0.142	0.81 [-0.45, 2.08]	0.86 [-0.32, 2.04]	0.150	-0.04 [-0.11, 0.03]	-0.04 [-0.15, 0.06]	0.403
Knee	0.00 [-0.43, 0.44]	0.22 [-0.85, 1.29]	0.681	0.22 [-0.32, 0.75]	0.49 [-0.35, 1.33]	0.246	-0.10 [-0.45, 0.25]	0.23 [-0.86, 1.32]	0.668	0.08 [-0.06, 0.22]	0.16 [-0.09, 0.40]	0.204

Ankle	-0.05 [-0.83, 0.74]	-0.23 [-0.89, 0.43]	0.489	-0.15 [-0.77, 0.48]	-0.35 [-1.42, 0.72]	0.513	-0.17 [-0.33, -0.00]	-0.15 [-0.62, 0.31]	0.505	0.26 [0.12, 0.40]	0.28 [0.02, 0.54]	0.034 *
**Transverse**												
Trunk	-0.06 [-0.35, 0.24]	-0.15 [-0.42, 0.13]	0.290	0.06 [-0.85, 0.97]	0.04 [-1.03, 1.12]	0.936	0.05 [-0.23, 0.33]	0.16 [-0.11, 0.42]	0.234	-0.00 [-0.01, 0.00]	0.00 [-0.00, 0.01]	0.310
Pelvis	0.24 [-0.43, 0.90]	0.47 [-0.42, 1.35]	0.289	-0.05 [-0.87, 0.76]	-0.19 [-1.20, 0.82]	0.710	-0.13 [-0.37, 0.11]	-0.32 [-1.00, 0.37]	0.356	0.00 [-0.00, 0.00]	0.00 [-0.00, 0.01]	0.349
Hip	0.40 [-0.17, 0.98]	0.27 [-0.56, 1.09]	0.511	0.55 [-0.16, 1.27]	0.70 [-0.11, 1.51]	0.088	-0.04 [-0.96, 0.89]	0.39 [-0.49, 1.28]	0.374	-0.11 [-0.24, 0.03]	-0.08 [-0.30, 0.15]	0.494
Foot Progression	0.90 [0.43, 1.37]	0.38 [-0.57, 1.34]	0.418	1.06 [0.16, 1.95]	0.84 [0.03, 1.64]	0.042 *	0.40 [0.10, 0.71]	0.50 [-0.28, 1.28]	0.200	0.01 [-0.15, 0.18]	-0.05 [-0.31, 0.21]	0.706
**Hip**												
**Sagittal**												
Trunk	-0.19 [-0.66, 0.28]	-0.39 [-1.17, 0.39]	0.324	0.08 [-0.43, 0.58]	-0.12 [-0.88, 0.63]	0.749	0.13 [-0.21, 0.48]	-0.20 [-0.91, 0.50]	0.566	0.01 [-0.00, 0.01]	0.01 [0.00, 0.02]	0.031 *
Pelvis	0.34 [-0.09, 0.77]	-0.34 [-1.16, 0.48]	0.409	-0.06 [-0.43, 0.32]	-0.80 [-1.54, -0.06]	0.034 *	0.37 [0.03, 0.71]	0.49 [-0.10, 1.07]	0.101	0.00 [-0.00, 0.00]	0.00 [-0.00, 0.00]	0.877
Hip	-0.00 [-0.02, 0.02]	-0.01 [-0.05, 0.03]	0.522	0.03 [-0.04, 0.10]	0.12 [0.01, 0.23]	0.034 *	0.05 [-0.05, 0.15]	0.09 [-0.04, 0.23]	0.181	0.01 [-0.01, 0.03]	0.01 [-0.02, 0.05]	0.398
Knee	0.00 [-0.04, 0.05]	-0.04 [-0.12, 0.05]	0.366	0.01 [-0.02, 0.04]	0.05 [-0.01, 0.10]	0.085	-0.04 [-0.16, 0.07]	-0.03 [-0.20, 0.14]	0.737	-0.02 [-0.04, 0.00]	-0.00 [-0.04, 0.03]	0.902

Ankle	0.04 [-0.24, 0.32]	0.20 [-0.25, 0.66]	0.368	-0.02 [-0.07, 0.02]	0.01 [-0.06, 0.08]	0.728	0.05 [-0.03, 0.12]	0.13 [0.00, 0.26]	0.044 *	0.01 [-0.01, 0.03]	0.02 [-0.02, 0.06]	0.343
Tibial Inclination	0.14 [-0.10, 0.38]	0.22 [-0.27, 0.71]	0.368	0.00 [-0.02, 0.03]	0.03 [-0.01, 0.07]	0.117	0.00 [-0.02, 0.02]	0.01 [-0.01, 0.04]	0.321	-0.00 [-0.02, 0.02]	0.02 [-0.01, 0.05]	0.193
Foot	-0.27 [-0.68, 0.15]	-0.20 [-0.80, 0.40]	0.501	0.09 [-0.06, 0.23]	0.16 [-0.07, 0.39]	0.160	0.02 [-0.01, 0.04]	0.04 [0.00, 0.07]	0.040 *	-0.00 [-0.02, 0.02]	-0.00 [-0.03, 0.02]	0.812
**Coronal**												
Trunk	0.03 [-0.02, 0.07]	0.10 [0.01, 0.19]	0.034 *	0.01 [-0.02, 0.04]	-0.02 [-0.08, 0.04]	0.502	-0.01 [-0.06, 0.04]	-0.08 [-0.16, 0.01]	0.068	-0.00 [-0.01, 0.01]	-0.02 [-0.04, 0.00]	0.124
Pelvis	0.03 [-0.06, 0.13]	0.03 [-0.10, 0.15]	0.675	0.01 [-0.06, 0.08]	0.04 [-0.05, 0.14]	0.380	-0.08 [-0.16, 0.01]	-0.07 [-0.19, 0.04]	0.220	0.00 [-0.01, 0.02]	0.01 [-0.01, 0.03]	0.327
Hip	-0.48 [-0.95, -0.00]	-1.02 [-1.85, -0.20]	0.016 *	-0.41 [-0.76, -0.06]	-0.28 [-0.81, 0.26]	0.304	-0.25 [-0.66, 0.15]	-0.21 [-0.88, 0.46]	0.534	0.01 [-0.03, 0.05]	-0.01 [-0.08, 0.06]	0.808
Knee	0.27 [-0.18, 0.72]	0.60 [-0.19, 1.39]	0.132	0.19 [-0.17, 0.55]	0.45 [-0.15, 1.06]	0.138	0.19 [-0.16, 0.54]	0.49 [-0.21, 1.20]	0.165	-0.02 [-0.13, 0.09]	0.07 [-0.11, 0.24]	0.462
Ankle	0.03 [-0.34, 0.40]	-0.34 [-0.86, 0.17]	0.191	-0.04 [-0.37, 0.29]	-0.05 [-0.73, 0.62]	0.873	0.23 [-0.06, 0.51]	0.23 [-0.21, 0.67]	0.302	-0.14 [-0.26, -0.03]	-0.11 [-0.30, 0.08]	0.237
**Transverse**												
Trunk	0.05 [-0.22, 0.32]	-0.04 [-0.40, 0.32]	0.826	0.26 [-0.21, 0.72]	0.32 [-0.43, 1.08]	0.396	-0.09 [-0.31, 0.12]	0.04 [-0.26, 0.33]	0.811	-0.00 [-0.00, -0.00]	0.00 [-0.00, 0.00]	0.242
Pelvis	0.15 [-0.38, 0.68]	0.42 [-0.38, 1.21]	0.298	-0.01 [-0.40, 0.39]	-0.29 [-0.96, 0.38]	0.395	0.01 [-0.49, 0.51]	0.04 [-0.69, 0.77]	0.910	0.00 [-0.01, 0.01]	0.00 [-0.01, 0.01]	0.850

Hip	-0.08 [-0.48, 0.31]	-0.20 [-0.84, 0.44]	0.535	0.37 [0.05, 0.69]	0.59 [0.06, 1.12]	0.031 *	0.28 [-0.01, 0.57]	0.63 [0.13, 1.13]	0.015 *	0.07 [-0.02, 0.15]	0.09 [-0.06, 0.24]	0.228
Foot Progression	-0.18 [-0.53, 0.17]	-0.55 [-1.20, 0.11]	0.102	-0.10 [-0.39, 0.19]	-0.23 [-0.71, 0.25]	0.336	-0.18 [-0.56, 0.20]	-0.12 [-0.74, 0.50]	0.704	0.01 [-0.10, 0.11]	-0.04 [-0.22, 0.14]	0.657
**Knee**												
**Sagittal**												
Trunk	-0.10 [-0.57, 0.37]	-0.15 [-0.99, 0.69]	0.723	0.01 [-0.34, 0.37]	-0.14 [-0.78, 0.49]	0.655	0.11 [-0.23, 0.45]	-0.20 [-0.92, 0.52]	0.586	-0.01 [-0.01, 0.00]	0.00 [-0.01, 0.01]	0.696
Pelvis	-0.68 [-0.97, -0.39]	-1.16 [-1.88, -0.44]	0.002 †	1.13 [0.70, 1.56]	0.20 [-0.62, 1.01]	0.635	-0.94 [-1.33, -0.56]	-1.00 [-1.68, -0.32]	0.005 †	0.00 [-0.00, 0.00]	-0.00 [-0.00, 0.00]	0.614
Hip	0.01 [-0.01, 0.03]	0.00 [-0.04, 0.04]	0.911	0.02 [-0.03, 0.06]	0.10 [-0.00, 0.19]	0.058	-0.05 [-0.21, 0.11]	-0.06 [-0.31, 0.19]	0.630	-0.02 [-0.03, 0.00]	-0.02 [-0.05, 0.02]	0.316
Knee	-0.01 [-0.05, 0.04]	-0.05 [-0.14, 0.04]	0.240	0.00 [-0.03, 0.03]	0.01 [-0.04, 0.06]	0.589	0.10 [0.03, 0.17]	0.15 [0.01, 0.28]	0.031 *	-0.00 [-0.02, 0.01]	0.02 [-0.01, 0.04]	0.309
Ankle	0.09 [-0.22, 0.40]	0.17 [-0.36, 0.71]	0.520	0.10 [0.05, 0.14]	0.14 [0.06, 0.21]	0.001 ‡	-0.15 [-0.24, -0.05]	-0.03 [-0.19, 0.14]	0.756	0.03 [-0.00, 0.05]	0.04 [-0.02, 0.09]	0.171
Tibial Inclination	0.05 [-0.26, 0.37]	0.32 [-0.28, 0.92]	0.297	-0.01 [-0.03, 0.01]	-0.01 [-0.04, 0.03]	0.766	0.01 [-0.01, 0.03]	0.02 [-0.01, 0.05]	0.174	0.02 [0.00, 0.05]	0.04 [0.01, 0.08]	0.014 *
Foot	0.30 [0.04, 0.55]	0.40 [-0.07, 0.86]	0.094	-0.37 [-0.51, -0.24]	-0.39 [-0.63, -0.15]	0.002 †	-0.02 [-0.04, -0.01]	-0.01 [-0.04, 0.03]	0.751	-0.01 [-0.02, 0.01]	-0.01 [-0.03, 0.02]	0.572
**Coronal**												

Trunk	-0.05 [-0.15, 0.06]	-0.02 [-0.19, 0.15]	0.839	0.01 [-0.03, 0.05]	0.01 [-0.06, 0.07]	0.781	-0.03 [-0.19, 0.14]	0.02 [-0.23, 0.27]	0.875	0.01 [0.00, 0.02]	-0.00 [-0.02, 0.02]	0.881
Pelvis	-0.11 [-0.21, -0.02]	-0.13 [-0.27, 0.02]	0.095	0.20 [-0.06, 0.47]	0.27 [-0.13, 0.66]	0.188	0.20 [0.08, 0.32]	0.24 [0.06, 0.42]	0.008 †	-0.00 [-0.01, 0.01]	-0.00 [-0.02, 0.02]	0.995
Hip	1.35 [1.01, 1.69]	0.84 [0.12, 1.57]	0.023 *	0.93 [0.62, 1.23]	0.83 [0.29, 1.37]	0.003 †	0.31 [-0.11, 0.73]	0.06 [-0.69, 0.82]	0.865	0.00 [-0.04, 0.04]	0.02 [-0.05, 0.09]	0.549
Knee	0.25 [-0.01, 0.50]	0.53 [-0.09, 1.15]	0.090	0.19 [-0.09, 0.47]	0.49 [-0.06, 1.04]	0.083	-0.36 [-0.60, -0.12]	-0.30 [-0.90, 0.30]	0.321	0.09 [0.01, 0.17]	0.20 [0.05, 0.36]	0.012 *
Ankle	-0.03 [-0.33, 0.27]	-0.30 [-0.79, 0.20]	0.240	-0.07 [-0.46, 0.31]	-0.37 [-1.10, 0.36]	0.319	-0.24 [-0.43, -0.06]	-0.27 [-0.62, 0.08]	0.124	0.22 [0.15, 0.29]	0.26 [0.10, 0.41]	0.001 †
**Transverse**												
Trunk	0.05 [-0.12, 0.22]	-0.01 [-0.29, 0.27]	0.954	0.08 [-0.36, 0.51]	-0.20 [-0.95, 0.55]	0.604	-0.07 [-0.24, 0.11]	0.01 [-0.27, 0.29]	0.964	0.00 [-0.00, 0.00]	0.00 [-0.00, 0.01]	0.104
Pelvis	0.07 [-0.30, 0.45]	0.30 [-0.37, 0.97]	0.378	0.25 [-0.17, 0.68]	0.08 [-0.67, 0.84]	0.827	0.18 [-0.11, 0.46]	0.05 [-0.48, 0.59]	0.841	0.00 [-0.00, 0.01]	0.01 [-0.00, 0.01]	0.109
Hip	0.33 [0.08, 0.58]	0.26 [-0.25, 0.78]	0.308	0.01 [-0.17, 0.19]	0.10 [-0.31, 0.51]	0.620	-0.09 [-0.43, 0.25]	0.23 [-0.37, 0.83]	0.452	-0.05 [-0.14, 0.03]	0.04 [-0.12, 0.19]	0.655
Foot Progression	0.21 [-0.15, 0.57]	-0.23 [-0.93, 0.47]	0.515	0.16 [-0.20, 0.52]	0.11 [-0.50, 0.72]	0.724	0.14 [-0.22, 0.50]	0.16 [-0.49, 0.81]	0.621	0.02 [-0.05, 0.09]	-0.01 [-0.16, 0.14]	0.893
**Foot & Ankle**												
**Sagittal**												

Trunk	-0.08 [-0.52, 0.35]	-0.38 [-1.11, 0.35]	0.304	-0.63 [-1.49, 0.23]	-0.95 [-1.94, 0.05]	0.061	-0.42 [-1.09, 0.26]	-0.85 [-1.81, 0.10]	0.078	-0.00 [-0.01, 0.01]	0.00 [-0.01, 0.01]	0.471
Pelvis	-0.78 [-1.28, -0.28]	-1.21 [-2.11, -0.31]	0.010 †	-0.65 [-1.18, -0.12]	-1.29 [-2.15, -0.43]	0.004 †	0.66 [-0.01, 1.33]	0.67 [-0.16, 1.50]	0.113	0.00 [-0.00, 0.00]	-0.00 [-0.00, 0.00]	0.986
Hip	-0.02 [-0.05, 0.01]	-0.04 [-0.09, 0.01]	0.083	0.04 [-0.02, 0.09]	0.11 [0.01, 0.21]	0.035 *	0.05 [-0.04, 0.15]	0.11 [0.00, 0.22]	0.049 *	0.01 [-0.02, 0.03]	0.01 [-0.02, 0.04]	0.707
Knee	-0.04 [-0.13, 0.04]	-0.08 [-0.19, 0.03]	0.155	0.01 [-0.02, 0.05]	0.04 [-0.01, 0.09]	0.097	-0.04 [-0.15, 0.06]	-0.04 [-0.19, 0.11]	0.607	-0.01 [-0.03, 0.01]	0.01 [-0.02, 0.04]	0.601
Ankle	0.31 [-0.28, 0.90]	0.49 [-0.21, 1.19]	0.169	-0.00 [-0.07, 0.06]	0.04 [-0.05, 0.13]	0.359	-0.07 [-0.24, 0.10]	0.03 [-0.18, 0.23]	0.804	-0.02 [-0.05, 0.02]	-0.01 [-0.06, 0.04]	0.756
Tibial Inclination	-0.05 [-0.42, 0.32]	0.04 [-0.55, 0.62]	0.904	-0.01 [-0.04, 0.03]	0.01 [-0.03, 0.06]	0.571	0.01 [-0.01, 0.03]	0.02 [-0.01, 0.05]	0.156	0.02 [-0.01, 0.04]	0.03 [-0.01, 0.06]	0.109
Foot	-0.03 [-0.34, 0.27]	0.00 [-0.45, 0.45]	0.996	-0.05 [-0.39, 0.29]	-0.02 [-0.41, 0.37]	0.924	-0.01 [-0.05, 0.03]	0.01 [-0.03, 0.05]	0.673	0.01 [-0.01, 0.03]	0.01 [-0.02, 0.03]	0.598
**Coronal**												
Trunk	-0.01 [-0.18, 0.15]	0.06 [-0.14, 0.25]	0.565	-0.03 [-0.06, 0.01]	-0.03 [-0.09, 0.02]	0.263	-0.03 [-0.11, 0.05]	-0.06 [-0.17, 0.05]	0.272	-0.02 [-0.04, 0.00]	-0.03 [-0.06, -0.01]	0.020 *
Pelvis	-0.02 [-0.13, 0.09]	-0.04 [-0.17, 0.08]	0.498	-0.08 [-0.19, 0.03]	-0.09 [-0.21, 0.03]	0.144	0.07 [-0.15, 0.30]	0.09 [-0.15, 0.32]	0.467	0.01 [-0.00, 0.02]	0.01 [-0.01, 0.03]	0.198
Hip	-0.62 [-1.24, 0.01]	-1.12 [-2.04, -0.19]	0.019 *	-0.43 [-0.75, -0.11]	-0.44 [-0.93, 0.04]	0.073	-0.19 [-0.56, 0.18]	-0.17 [-0.80, 0.46]	0.598	-0.00 [-0.05, 0.04]	-0.01 [-0.08, 0.06]	0.784

Knee	0.24 [-0.28, 0.76]	0.37 [-0.43, 1.18]	0.354	0.15 [-0.29, 0.59]	0.28 [-0.36, 0.92]	0.383	0.22 [-0.17, 0.61]	0.43 [-0.32, 1.19]	0.256	-0.14 [-0.25, -0.03]	-0.07 [-0.25, 0.10]	0.406
Ankle	0.13 [-0.21, 0.46]	-0.08 [-0.51, 0.36]	0.727	-0.08 [-0.89, 0.72]	0.04 [-1.01, 1.09]	0.946	0.20 [-0.16, 0.56]	0.24 [-0.23, 0.71]	0.302	-0.15 [-0.28, -0.02]	-0.14 [-0.34, 0.06]	0.155
**Transverse**												
Trunk	0.02 [-0.18, 0.21]	-0.04 [-0.28, 0.20]	0.733	0.14 [-0.61, 0.89]	0.21 [-0.74, 1.17]	0.657	-0.07 [-0.27, 0.13]	-0.01 [-0.25, 0.23]	0.952	0.00 [-0.00, 0.00]	0.00 [0.00, 0.01]	0.019 *
Pelvis	-0.13 [-0.67, 0.42]	0.08 [-0.65, 0.82]	0.821	0.15 [-0.35, 0.66]	-0.06 [-0.79, 0.68]	0.881	0.13 [-0.31, 0.58]	-0.01 [-0.62, 0.61]	0.977	0.00 [-0.01, 0.01]	0.00 [-0.01, 0.01]	0.584
Hip	-0.11 [-0.59, 0.38]	-0.22 [-0.89, 0.45]	0.514	0.08 [-0.21, 0.38]	0.21 [-0.29, 0.71]	0.397	-0.09 [-0.60, 0.42]	0.27 [-0.39, 0.93]	0.413	0.06 [-0.05, 0.18]	0.11 [-0.06, 0.28]	0.213
Foot Progression	-0.33 [-0.84, 0.18]	-0.83 [-1.57, -0.10]	0.027 *	-0.16 [-0.70, 0.38]	-0.33 [-0.99, 0.32]	0.313	0.33 [-0.09, 0.76]	0.36 [-0.28, 0.99]	0.261	0.02 [-0.11, 0.15]	-0.03 [-0.23, 0.16]	0.744

Sagittal, coronal, and transverse plane joint angles measured at initial contact, midstance, and toe-off, as well as range of motion (ROM) across the gait cycle, for runners with spine, hip, knee, or foot/ankle pathology and controls. Asymmetry index (AI) was calculated as log(Symptomatic / Asymptomatic). AI = 0 indicates symmetry; positive values indicate greater angular magnitude on the symptomatic limb; negative values indicate greater magnitude on the contralateral limb.
